# Farm-Level Biosecurity and Antimicrobial Use in Pig Production: An Integrated Inferential and Explainable Machine Learning Analysis

**DOI:** 10.3390/vetsci13090971

**Published:** 2026-09-16

**Authors:** Krisztián Vribék, Máté Farkas, Szilveszter Csorba, Miklós Süth, László Gombos, Ádám Kerek, Evelin Imre, Zoltán Somogyi, Ákos Jerzsele, Zsuzsa Farkas

**Affiliations:** 1 Department of Digital Food Science, Institute of Food Chain Science, University of Veterinary Medicine, H-1078 Budapest, Hungary; vribek.krisztian@univet.hu (K.V.); csorba.szilveszter@univet.hu (S.C.); evelinimre00@gmail.com (E.I.); farkas.zsuzsa@univet.hu (Z.F.); 2 National Laboratory of Infectious Animal Diseases, Antimicrobial Resistance, Veterinary Public Health and Food Chain Safety, University of Veterinary Medicine Budapest, H-1078 Budapest, Hungary; suth.miklos@univet.hu (M.S.); kerek.adam@univet.hu (Á.K.); somogyi.zoltan@univet.hu (Z.S.); jerzsele.akos@univet.hu (Á.J.); 3 Institute of Food Chain Science, University of Veterinary Medicine, H-1078 Budapest, Hungary; 4 Wittmann Antal Multidisciplinary Doctoral School of Plant, Animal and Food Sciences, Széchenyi István University, 2 Vár Square, H-9200 Mosonmagyaróvár, Hungary; drgomboslaci@gmail.com; 5 Department of Pharmacology and Toxicology, University of Veterinary Medicine, H-1078 Budapest, Hungary

**Keywords:** antimicrobial use, biosecurity practice, explainable machine learning, swine farming, veterinary public health, SHAP

## Abstract

Internal biosecurity may be associated with antimicrobial use (AMU) in pig production, but observational associations, predictions and explanations of fitted models answer different questions. We analysed 1110 farm–month–age-group units from 18 Hungarian pig farms; an original post hoc restriction retained 939 units from 13 farms. The continuous endpoint was the natural logarithm of one plus a defined AMU index formed by summing substance-specific active ingredient mass-to-recorded-weight ratios. The study-specific biosecurity instrument was used for within-farm monitoring and was not externally validated. We retained a 2 × 2 matrix of unfiltered/filtered and composite/separate-variable models, with categorical animal-group separation, age-group adjustment and farm-cluster uncertainty. In the unfiltered separate-variable model, recorded separation code 2 versus code 0 was associated with a higher log AMU index (β = 0.205; CR1-t 95% CI 0.013–0.397; *p* = 0.037), although the wild-cluster *p*-value was 0.116 and sensitivity estimates varied with adjustment. Code 3 binary contrasts were too sparse for precise effect quantification. Four ML families were evaluated using nested farm-grouped and leave-one-farm-out validation. For the inner-selected biosecurity-plus-age procedure, the unfiltered pooled R^2^ was −0.101 and −0.187, respectively. SHAP retained an exploratory role in explaining held-out tree model predictions. The results identify model-dependent observational patterns but do not establish a causal biosecurity effect or reliable prediction for new farms.

## Simple Summary

Antimicrobial resistance is a growing threat to both human and animal health. The use of antimicrobial medicines in livestock can contribute to this problem, making it important to understand how farm management can help reduce the need for treatment. This study examined whether biosecurity practices on pig farms were related to the amount of antimicrobials used. Particular attention was given to measures that limit contact between different groups of animals and reduce the introduction and spread of disease. Antimicrobial use varied considerably between farms and between different stages of pig production. Poorer separation of animal groups was generally associated with higher antimicrobial use, although the relationship was not always straightforward and was influenced by conditions on individual farms. Hygiene-related practices also appeared to contribute, but their effects were less clearly linked to antimicrobial use when considered separately. The analysis found that associations between recorded animal-group separation and antimicrobial use depended on the way other hygiene measures and farm context were represented. The original continuous model estimates were retained, but additional checks showed that the strength of evidence was sensitive to the method used to account for the small number of farms. Tree-based explanations, including SHAP, describe how the fitted models used the recorded variables. Validation on farms excluded from training did not establish a reliable predictive benefit from biosecurity beyond production stage information and simple baselines. These findings support cautious interpretation and further validation rather than direct use as a farm-level risk prediction tool.

## 1. Introduction

Antimicrobial resistance (AMR) is widely recognised as one of the most pressing One Health challenges, threatening the effectiveness of antimicrobial therapy in human and veterinary medicine and affecting the interconnected domains of animal, human, and environmental health worldwide [1]. In livestock production, increasing global demand for animal protein has contributed to intensification, particularly in pig farming [2], where high stocking densities, rapid turnover, and continuous production cycles may promote frequent antimicrobial use (AMU) [3] and thereby increase selective pressure for resistance [4]. Among food-producing animals, pigs are one of the most intensively treated species, especially during early life and around weaning, when physiological stress, immature immune function, and disruption of intestinal barrier integrity increase susceptibility to enteric and systemic disease [5,6,7,8]. Consequently, pig production systems represent an important setting for antimicrobial stewardship, both because of their contribution to veterinary AMU and because of their relevance to broader AMR mitigation efforts. Reflecting this, international and European policy frameworks have increasingly prioritised reductions in veterinary AMU within broader AMR action plans and stewardship initiatives [9,10].

Sustainable reduction of AMU in livestock, however, cannot rely on restriction alone. It also requires a better understanding of the farm-level conditions under which antimicrobial demand arises [11,12]. Recent evidence from Dutch pig farms indicates that respiratory, musculoskeletal, and neurological diseases, particularly in weaners, are important drivers of antimicrobial group treatments, while the occurrence of these conditions is itself associated with farm-level management, biosecurity, housing, and preventive health characteristics [13]. Among the candidate determinants, farm management practices, especially biosecurity [14] and preventive health measures such as vaccination, are widely regarded by veterinarians and animal health professionals as central influences on herd health and treatment pressure [15]. Several studies have reported associations between improved biosecurity, better management, or more favourable housing conditions and lower AMU in pig production [16,17,18]. Nevertheless, quantitative evidence linking specific biosecurity measures to AMU at the farm level remains limited and methodologically challenging to interpret [19,20,21]. Farm-level observational datasets are often heterogeneous, contain correlated management indicators, and reflect complex farm-specific contexts. In practice, biosecurity measures are rarely implemented as isolated interventions; rather, they tend to cluster as components of broader management packages. Consistent with this complexity, a longitudinal multi-method analysis of Dutch pig farms found that grouping related farm characteristics into management domains provided additional insight beyond conventional risk-factor modelling, with internal biosecurity showing the greatest overall contribution to variation in AMU [22]. As a result, simple pairwise relationships may underestimate the contribution of biosecurity, while collinearity and interaction structures may obscure which features are most strongly associated with AMU differences between farms [16,19,20].

At the same time, recent developments in farm-level digitalisation have created new opportunities for data-driven approaches in livestock epidemiology. Detailed AMU records, standardised biosecurity scoring systems [23,24,25] and routine monitoring have increased the availability of longitudinal real-world production and other routine farm data [26]. These data environments are well suited to machine learning (ML) approaches, which can detect non-linear patterns, interactions, and locally important predictor structures that conventional linear methods may fail to capture [27]. Previous research has similarly demonstrated the value of combining complementary analytical approaches [22]. However, in applied animal health research, predictive performance alone is insufficient; analytical frameworks must also remain interpretable to support trust, transparency, and practical uptake among veterinarians, producers, and regulators [28,29]. Post hoc explainability methods, including SHapley Additive exPlanations (SHAP) [30], offer a way to decompose complex model outputs into understandable feature contributions and thereby provide both global and local explanations of model behaviour [31]. Such approaches have already shown value in livestock-related applications, including the interpretation of movement-derived cattle behaviour patterns and sensor-based physiological predictions in dairy cows [32,33]. In swine systems, post hoc explainability methods have also been used to rank disease risk and quantify the relative contribution of biosecurity-related factors to outbreak probability [27,34]. These methods therefore offer a promising route for translating complex farm record data into actionable and biologically meaningful evidence.

**Figure 1:**
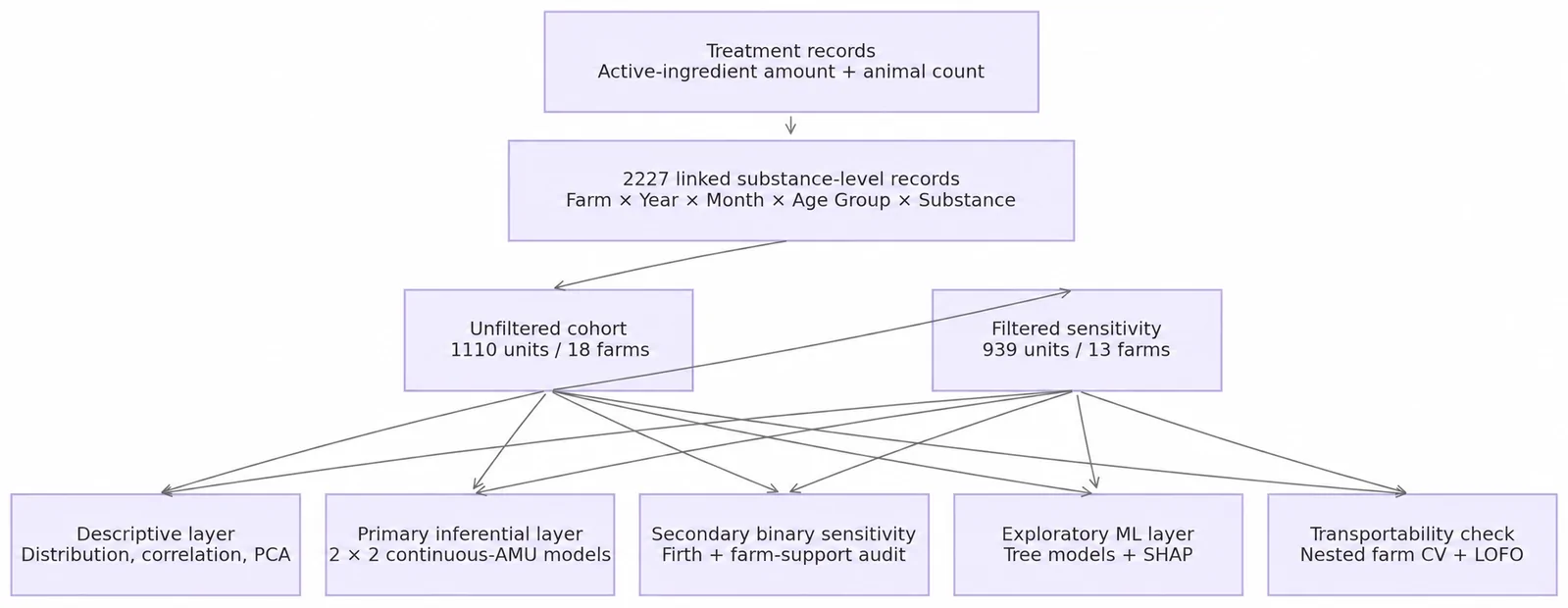
Analytical workflow and cohort structure. Source pig farm records containing synchronous antimicrobial use and biosecurity measurements were aggregated to the Year × Month × Farm × Age Group level. The unfiltered cohort was retained as the primary analytical population, while the restricted filtered cohort was evaluated as a post hoc sensitivity population. Analyses were organised into descriptive, primary inferential, secondary binary sensitivity, exploratory explainable machine learning, and farm-held-out transportability layers.

The present study was designed to quantify the relationship between farm-level biosecurity practices and AMU in pig production systems using an integrated inferential and explainable ML framework. Specifically, the study was based on real-world pig farm data to examine how biosecurity measures relate to AMU when represented either as individual ordinal variables or as a composite hygiene deficit index. In parallel, we applied explainable ML methods to evaluate predictor importance and characterise non-linear feature effects, while inferential statistical models were used to estimate associations and assess robustness across alternative cohort definitions and variable representations. By integrating these complementary analytical perspectives, the study aimed to provide a more transparent and data-driven basis for understanding AMU variation in pig farming and to support the development of more targeted antimicrobial stewardship strategies. We asked whether recorded animal-group separation and broader hygiene conditions were associated with continuous AMU after age-group adjustment; whether these associations were robust to alternative biosecurity representations, cohort restrictions and farm influence; and whether the available variables improved prediction in farms excluded from model training. Poorer separation and hygiene provide a biological motivation for expecting greater AMU, but this is not presented as a preregistered hypothesis. Conventional regression addresses adjusted association, grouped validation addresses prediction, and SHAP describes the behaviour of fitted tree models. The contribution is their explicit separation within one farm record study, with the analytical hierarchy shown in Figure 1.

## 2. Materials and Methods

### 2.1. Study Design and Analytical Objective

This observational study investigated the association between farm-level biosecurity measures and antimicrobial use (AMU) in pig production using an integrated epidemiological and explainable machine learning (ML) workflow (Figure 1). The analytical units were Farm × Year × Month × Age Group combinations, representing repeated monthly production-stage observations nested within farms. The analysis followed the following analytical hierarchy: continuous outcome inferential models formed the primary analytical layer, while binary high-AMU models and machine learning analyses served as secondary and exploratory components.

The primary analytical objective was to quantify associations between biosecurity conditions and continuous AMU, expressed as the natural logarithm of one plus the sum-of-ratios AMU index defined in Section 2.2. Animal Population Separation was treated as the main biosecurity exposure and modelled categorically. The remaining eight hygiene- and management-related biosecurity variables were evaluated either as separate numerically coded ordinal predictors or through their unweighted mean, termed the mean coded hygiene score. This allowed a comparison between a parsimonious composite specification and a more detailed separate-variable specification. The parameter-specific category definitions and coding direction are provided in Table S1; the assumptions associated with the numerical representations are described in Section 2.4.

Analyses were conducted in both an unfiltered cohort and a restricted filtered cohort. The unfiltered cohort served as the primary analysis set. The filtered cohort was evaluated as a restricted population sensitivity analysis because the exclusions changed the target population and were therefore not interpreted as simple data cleaning. The cohort restrictions and additional reviewer-requested sensitivity analyses were developed after data inspection and were not preregistered. Comparisons across cohort definitions were used to assess the robustness of the observed associations while preserving the unfiltered data as the main basis for inference.

The analytical workflow consisted of five layers. First, descriptive analyses characterised the AMU distribution, farm-level heterogeneity, correlation structure and effects of cohort restriction. Second, primary inferential models estimated associations between biosecurity variables and the continuous log-transformed AMU index, accounting for clustering within farms when estimating uncertainty. Third, sensitivity analyses examined alternative biosecurity representations and adjustment specifications, cohort restriction, leave-one-farm-out influence and wild-cluster bootstrap uncertainty. Fourth, binary high-AMU models were fitted as secondary sensitivity analyses using Firth bias-reduced logistic regression because some exposure strata had sparse outcome support or complete separation. Fifth, exploratory machine learning analyses examined multivariable predictive relationships and model behaviour, while farm-held-out cross-validation assessed prediction in farms excluded from training. SHAP summaries were interpreted as explanations of fitted-model predictions rather than causal effect estimates or independent evidence of predictive utility.

### 2.2. Source Data and Unit of Analysis

The source dataset contained antimicrobial use records, farm identifiers, age-group information, calendar time variables and ordinal biosecurity measurements from commercial pig farms in Hungary. The linked preprocessing export comprised 2227 active-substance-level records from 18 farms, covering recorded treatment-start months from November 2021 to November 2025. Biosecurity measurements described hygiene, separation and equipment management conditions in the corresponding production units.

During preprocessing, treatment records were assigned to calendar year and month according to the recorded treatment start date and grouped by Farm × Year × Month × Age Group × Active Substance. Active ingredient amount and animal count were summed within each group. The aggregated active ingredient amount was then divided by the aggregated animal count multiplied by an age-group-specific reference body weight: 65 kg for fattening and pre-fattening pigs, 25 kg for post-weaning pigs, 3.5 kg for suckling piglets, and 240 kg for sow and breeding gilt categories. This calculation began from recorded active ingredient quantity, rather than an already normalised mg/kg variable. Active substance remained part of the preprocessing aggregation key, so different substances used within the same farm–month–age-group combination remained separate records.

For analysis, these substance-level records were aggregated to Farm × Year × Month × Age Group units. The analytical export already contained the substance-specific normalised amounts, expressed in mg/kg; these values were summed once within each analytical unit, without further normalisation. Let (M_{bs}) denote the aggregated active ingredient mass, expressed in mg, for substance (s) within analytical unit (b), (N_b) the recorded animal count, and (W_b) the reference body weight in kg. Because recorded animal counts were identical across substances within each unit, the retained sum-of-ratios AMU index was (Equation (1)):

(1) $$S_{b}=\sum_{S}\frac{M_{bs}}{N_{b}W_{b}}=\frac{\sum_{s}M_{bs}}{N_{b}W_{b}}\;[\mathrm{mg}/\mathrm{kg}]$$

The same animals could contribute to records for more than one substance and could be counted repeatedly. Consequently, (N_b) was not interpreted as a verified count of unique animals. The index quantifies recorded mass-based AMU; it does not measure treatment frequency or constitute a DDDvet-based indicator.

The primary outcome was the natural log-transformed AMU index (2):

(2) $$Y_{b}=\ln\left(1+\frac{S_{b}}{1\mathrm{mg}/\mathrm{kg}}\right)$$

This transformation was used to reduce right skewness while retaining the quantitative information in the continuous outcome. Although the transformation accommodates zero values, all AMU values in the available analytical export were positive. Months absent from the export were not assigned zero AMU.

Biosecurity information was linked to the corresponding monthly production stage units rather than treated as a fixed historical farm attribute. Each analytical unit was associated with one internal biosecurity assessment, and identical biosecurity values repeated across its substance-level records were retained once. No conflicting within-unit score vectors or tied modes occurred among the 1110 analytical units; therefore, no modal aggregation or tie-breaking rule affected exposure construction. The month-level linkage did not establish the precise temporal ordering of biosecurity assessment and antimicrobial treatment or capture changes occurring within a month.

The unfiltered analytical dataset contained 1110 farm–month–age-group units from 18 farms, corresponding to 301 observed farm months. These units, referred to as analytical batches, were defined by the aggregation keys and did not necessarily represent distinct biological cohorts of animals. Because repeated observations originated from the same farms across months and age groups, farm-level dependence was addressed through farm-clustered uncertainty estimates in the inferential analyses and farm-held-out grouped cross-validation in the predictive analyses. A restricted cohort of 939 units from 13 farms was retained as a sensitivity population, reflecting the exclusion of selected farms and production-stage categories rather than data cleaning. Binary high-AMU indicators were defined only for secondary sensitivity analyses using the median of the log-transformed AMU index within each analytical cohort.

The animal-related data included in this study originated from commercial pig farms operating in Hungary and were privately owned by the respective farm companies. No animals were subjected to experimental manipulation, additional handling beyond routine veterinary practice, or treatment specifically for the purposes of this research. The study did not involve invasive procedures. The study protocol was reviewed by the Animal Welfare Committee of the University of Veterinary Medicine Budapest, which issued a Certificate of Exemption confirming that the project does not constitute an animal experiment under Act XXVIII of 1998 on Animal Protection and Government Decree No. 40/2013 on animal experiments.

### 2.3. Biosecurity Measures

Internal biosecurity was assessed monthly using a farm-specific monitoring and decision support instrument. Nine parameters were recorded separately for production units: hand disinfection availability and quality, glove use, foot disinfection availability and quality, separation of animal groups, separation of personnel, separation of equipment, and equipment hygiene. The instrument used parameter-specific descriptive criteria in a 0–4 scoring framework; the assessor selected the criterion corresponding to the observed condition. For example, the supplied instrument description assigns hand/foot disinfection availability scores 0, 2 and 4 to availability at every room entrance, at some entrances and absence, respectively. The supplied separation endpoints are consistent with all-in/all-out management (instrument score 1) and continuous mixing (instrument score 4). These examples describe the instrument, not an assumed recoding of the analytical export.

Assessments were completed by a veterinarian or a trained farm caretaker, generally the department head, instructed in completion of the register. The same assessor was preferred over time to support consistency; compliance with the farm biosecurity control plan was the principal quality control check. The instrument was not externally validated or derived from Biocheck.UGent (Ghent University, Ghent, Belgium) or another standardised index. Scores are therefore interpreted as within-farm monitoring measures, with uncertain equivalence between farms.

The source audit confirmed one linked assessment per analytical unit. Identical scores repeated across substance rows were retained once per unit. No conflicting within-unit score vectors or tied modes occurred (0/1110 units; 0%); no modal aggregation or tie-breaking rule affected exposure construction. This does not establish that assessments were unique or independent across all units. Exact assessment dates and within-month changes were not available in the month-level export.

Animal-group separation was modelled categorically using recorded code 0 as the reference. The export uses codes 0–3; item-specific export-to-instrument mapping must be distinguished from the original instrument’s numeric ranges. We use neutral recorded-code labels and do not assume equally spaced biological effects.

### 2.4. Alternative Representations of Biosecurity

To examine how the representation of biosecurity information influenced the estimated associations with AMU, two alternative specifications were evaluated in parallel in the main inferential analyses. Both retained Animal Population Separation as the main exposure, modelled categorically, and included adjustment for age group.

In the composite specification, the remaining eight biosecurity variables were summarised by their unweighted row-wise mean, termed the mean coded hygiene score. These variables were hand disinfection availability, hand disinfection practice, glove use, foot disinfection availability, foot disinfection practice, personnel separation, equipment separation, and equipment condition and hygiene. Animal Population Separation was excluded from this score. The author-confirmed codebook specifies a common coding direction, with higher values indicating poorer recorded conditions (Table S1). Accordingly, higher mean coded hygiene scores represented a greater average recorded biosecurity deficit. However, the items have different ranges, and calculating their mean assumes that numerical code increments are commensurable across components. The score was therefore treated as an analytical summary rather than an externally validated biosecurity index.

In the separate-variable specification, the same eight variables were entered individually as numeric covariates using their original ordinal codes. This specification estimated component-specific associations conditional on Animal Population Separation, age group and the other included biosecurity variables. Although the codebook establishes the intended ordering of the categories, each numeric slope assumes a linear association per coded step on the model’s linear predictor scale. Categorical coding of the eight variables was therefore examined as a sensitivity analysis to this assumption.

Additional summaries based on principal component analysis, one-factor analysis and weighted averaging were retained as supplementary sensitivity representations. The weighted mean used weights of 1.0, 1.2, 0.8, 1.0, 1.2, 1.0, 1.0 and 1.2 for the eight variables in the order listed above. These weights were retained from the original analytical code as sensitivity choices; they were not validated weights or production unit weights derived from the scoring instrument. PCA and factor scores calculated from the full cohort were not used as precomputed predictors in farm-held-out validation.

The composite specification provided a parsimonious representation of the broader hygiene environment, whereas the separate-variable specification allowed its components to have different estimated associations with AMU. Comparing the two specifications also assessed whether the association between Animal Population Separation and AMU depended on how the remaining biosecurity variables were represented. Component-specific estimates were interpreted as adjusted observational associations, with attention to correlations among predictors and their category support.

### 2.5. Age-Group Structure

Age group was treated as a key structural covariate because antimicrobial use patterns differ substantially across pig production stages. Differences in physiology, disease susceptibility, management and treatment practices mean that production categories cannot be assumed to represent equal steps along a numerical scale. In the primary inferential analyses, age group was therefore modelled as a categorical factor. This avoided imposing an artificial linear ordering on biologically distinct production stages and allowed stage-specific differences in the log-transformed AMU index.

Age group was included as an adjustment variable in both the composite and separate-variable biosecurity specifications. The source category “utónevelt sertés” is referred to as post-weaning pigs in the manuscript and corresponds to the label “Post-nursery pig” retained in the figures. The reference body weight used for this category was 25 kg, as specified in Section 2.2.

Age-group structure was also relevant to cohort restriction. The unfiltered cohort included all available production-stage categories and was retained as the primary analytical population. The filtered cohort excluded selected categories and was interpreted as a restricted population sensitivity analysis. Unused factor levels were removed before model fitting so that each model matrix reflected the categories present in the corresponding analytical population.

### 2.6. Data Cleaning and Preprocessing

Input data were processed using a reproducible workflow that standardised column names, harmonised expected variable aliases, trimmed categorical fields and converted the AMU and biosecurity variables to numeric format. Calendar variables, farm identifiers, age-group labels, substance-specific normalised AMU values and biosecurity codes were checked before aggregation. The observed biosecurity codes were compatible with the parameter-specific ranges in the author-confirmed codebook (Table S1).

All retained analytical variables were complete. Consequently, no observations were excluded because of missing data, no complete-case selection was required and no imputation was performed in either the inferential or machine learning analyses. The absence of missing values applied to the available analytical records; it did not establish complete calendar coverage, and absent farm months were not imputed as zero AMU.

As described in Section 2.2, the analytical input already contained substance-specific normalised AMU values. These were summed once within each Farm × Year × Month × Age Group unit to construct the sum-of-ratios AMU index, which was then log-transformed. No second biomass normalisation was applied. Each analytical unit had one linked biosecurity assessment, with identical score values repeated across its substance-level records. These values were retained once per unit. No conflicting within-unit scores or tied modes occurred, and no modal aggregation or highest-category tie-breaking rule affected the analytical data.

All subsequent analyses used the resulting farm–month–age-group dataset. Within each cohort, sample sizes were identical across the retained biosecurity specifications: 1110 units in the unfiltered cohort and 939 units in the filtered cohort. This difference resulted from the stated cohort restrictions rather than variable-specific missingness. In the machine learning analyses, encoding and scaling were fitted within the corresponding training partitions, and model selection followed the grouped validation procedures described below.

### 2.7. Cohort Definitions and Filtering Strategy

The main inferential analyses followed a 2 × 2 scenario matrix defined by cohort and biosecurity representation:

• Unfiltered composite models;
• Unfiltered separate-variable models;
• Filtered composite models;
• Filtered separate-variable models

The unfiltered cohort was retained as the primary analytical population and the main basis for inference. A restricted filtered cohort was evaluated as a sensitivity population. The restriction excluded pregnant gilts, pregnant sows and pre-fattening pigs, together with records from five farms, reducing the dataset from 1110 to 939 analytical units and from 18 to 13 farms. Farm names were withheld to preserve confidentiality.

The restricted cohort was developed during the machine learning workflow development phase, following inspection of cohort heterogeneity, subgroup support and model stability. It was therefore retained as a post hoc restricted population sensitivity analysis rather than a prespecified primary population. Its purpose was to examine how the estimated associations changed under the historical restriction. The exclusions altered the target population and were not assumed to improve data quality, eliminate confounding or produce inherently more reliable estimates.

All conclusions prioritise the unfiltered cohort. Filtered cohort results were used to assess sensitivity to cohort composition, including changes in effect estimates, uncertainty and exposure support. Agreement between the analyses was considered consistency across related populations, rather than independent replication; discrepancies indicated that the estimated associations depended on cohort composition.

An exclusion audit and filter decomposition analysis documented the consequences of restriction. Applying only the production-stage exclusions retained 963 units from 18 farms; applying only the farm exclusions retained 1086 units from 13 farms; and applying both retained 939 units from 13 farms. The corresponding association estimates are reported in Table S2. These restrictions overlap, so the numbers excluded by the two components are not additive. The audit also summarised changes in farm and age-group composition and the distributions of AMU and biosecurity variables. Table 1 presents the two analytical populations used in the main scenario matrix. A summary of the analytical populations used throughout the study, including the number of aggregated farm–month–age-group batches and farms in the unfiltered and filtered cohorts, is provided.

### 2.8. Support Diagnostics

Support diagnostics characterised the distribution of Animal Population Separation and identified limitations arising from sparse observations or concentration within a small number of farms. For each separation category in each cohort, the audit reported the number of analytical units, the number of contributing farms and the proportion of units contributed by the largest contributing farm. For the secondary binary analyses, separation categories were cross-tabulated against the cohort-specific high-AMU indicator, reporting high- and low-AMU counts, the high-AMU proportion and its Wilson 95% confidence interval (Table S3).

Wilson intervals were treated as descriptive summaries based on an independent-binomial assumption. They do not account for repeated observations within farms and were not used as substitutes for farm-adjusted inferential uncertainty. The support summaries identified exposure categories with all or nearly all observations in one outcome class and informed interpretation of the Firth logistic sensitivity analyses.

To assess the feasibility of examining age-group modification of the separation–AMU association, all age-group × separation-category cells, including empty cells, were tabulated with their unit and farm counts (Table S4). During revision, a support screen was introduced requiring at least 10 analytical units from at least three farms in every cell for the full interaction analysis. This was an analytical screening choice rather than a universal adequacy criterion, and meeting it would not by itself guarantee reliable inference. Stage-specific models were considered where the corresponding separation categories met the support screen and the model design matrix had full rank; available estimates are reported in Table S5. Failure to meet the screen was interpreted as insufficient support for the proposed analysis, not evidence that effect modification was absent.

Support diagnostics were considered alongside the restriction audit because excluding farms and production stages changed the observations and farm representation available for each separation category. These checks guided interpretation of both continuous and binary estimates, while continuous outcome models remained the primary inferential basis.

### 2.9. Inferential Statistical Analysis

#### 2.9.1. Primary Continuous Outcome Models

The primary inferential analysis modelled the continuous log-transformed AMU index defined in Equation (1) using ordinary least squares (OLS) regression. The continuous endpoint was retained because it preserved quantitative information in the antimicrobial use records and avoided the information loss introduced by dichotomisation. Animal Population Separation was modelled categorically, with code 0, representing consistently implemented strict all-in/all-out management, as the reference category. This specification did not impose a linear or monotonic association across separation categories.

Models followed the 2 × 2 cohort-by-biosecurity-specification matrix described in Section 2.7. Each model included categorical age group and either the mean coded hygiene score or the eight separate numerically coded hygiene variables. The unfiltered cohort remained the primary analytical population, with the filtered cohort providing a restricted population sensitivity analysis.

Dependence among observations from the same farm was addressed using farm-cluster-robust CR1 covariance estimates. The finite-sample covariance correction was [G/(G − 1)] × [(n − 1)/(n − *p*)], where G denotes the number of farms, n the number of analytical units and *p* the number of estimated regression parameters, including the intercept. Conventional coefficient tests and 95% confidence intervals used a Student’s t distribution with G − 1 degrees of freedom. The unfiltered and filtered cohorts contained 18 and 13 farms, respectively.

Because the number of farms was limited, inference was supplemented by null-imposed, studentized wild-cluster bootstrap tests using 9999 Rademacher draws. Each draw assigned a common weight of −1 or +1 to observations within the same farm. Tests evaluated the individual Animal Population Separation contrasts and the joint null hypothesis that all three separation coefficients were zero. Wild-cluster *p*-values were reported alongside the conventional CR1-t results. The bootstrap tests and CR1-t confidence intervals are distinct inferential procedures and were not assumed to yield identical conclusions.

Model diagnostics included residual, quantile–quantile and scale–location plots, leverage and Cook’s distance summaries, and leave-one-farm-out influence analyses. Additional sensitivity specifications examined categorical coding of the hygiene variables, calendar adjustment, farm-specific intercepts, alternative composite representations and the historical cohort restrictions. Farm-specific intercepts were used to assess sensitivity to persistent differences between farms; they were not interpreted as eliminating all confounding or establishing causal effects of within-farm changes.

A further sensitivity analysis used a Poisson quasi-likelihood model with a log link to examine associations with the conditional arithmetic mean of the untransformed AMU index, using farm-clustered uncertainty estimates. This model targeted a different outcome scale from the primary log-transformed OLS analysis and did not assume that antimicrobial mass was a count. Because the available export contained no zero AMU values, an any-use or zero-inflation component was not estimated. The continuous outcome estimates, their uncertainty and the sensitivity analyses jointly informed the interpretation of the observed biosecurity–AMU associations.

#### 2.9.2. Secondary Binary Sensitivity Analysis

A secondary binary endpoint, termed High Usage Class, identified analytical units whose log-transformed AMU index exceeded the median within the corresponding cohort (Equation (3)). The thresholds were 0.752072054 for the unfiltered cohort and 0.815935145 for the filtered cohort. Values equal to or below the threshold were assigned to the low-AMU category. The same cohort-specific threshold was used for both the composite and separate-variable specifications. This endpoint described relatively high AMU within each cohort rather than exceedance of a clinical or regulatory threshold.

(3) $$H_{b}=\left\{\begin{array}{c} 1,\;Y_{b}>m_{c},\\ 0,\;Y_{b}\leq m_{c}, \end{array}\right.\;m_{c}=median\left\{Y_{j}:j\in c\right\}.$$

Here, $H_{b}$ denotes the High Usage Class for analytical unit b, and $m_{c}$ is the median within its analytical cohort c.

The binary endpoint was retained only for sensitivity analysis because dichotomisation reduces information relative to the continuous outcome. Support diagnostics identified sparse observations and complete or quasi-complete separation in some Animal Population Separation strata, particularly after cohort restriction. Binary associations were therefore estimated using Firth bias-reduced logistic regression. Animal Population Separation was modelled categorically, with code 0 as the reference, together with categorical age group and either the mean coded hygiene score or the eight separate numeric hygiene covariates. Odds ratios were obtained by exponentiating the fitted separation coefficients.

Firth penalisation addresses bias and separation in logistic estimation but does not itself account for dependence among observations from the same farm. Uncertainty was therefore assessed using a whole-farm pairs bootstrap with 1999 draws. Farms were sampled with replacement, retaining all analytical units belonging to each sampled farm, and the corresponding model was refitted. The original cohort-specific AMU threshold was held fixed throughout resampling.

Estimability and convergence were audited across bootstrap draws. Exploratory 95% percentile confidence intervals were reported only when at least 90% of the requested draws were estimable; otherwise, intervals were withheld and the number of successful draws was reported alongside the point estimates. This reporting screen did not guarantee reliable inference in sparsely supported categories, and the resulting intervals were interpreted alongside the number of contributing farms and the concentration of observations within farms.

For descriptive comparison across cohorts, high- and low-AMU counts based on the common unfiltered-cohort median were also reported separately. These summaries did not replace the cohort-specific thresholds used in the binary models. Binary results were interpreted as secondary evidence about associations with relatively high AMU, rather than causal risk estimates or a replacement for the primary continuous outcome analysis.

### 2.10. Explainable Machine Learning Analysis

Machine learning analyses complemented the primary inferential models by examining multivariable predictive relationships, non-linear model behaviour and prediction in farms excluded from training. The workflow distinguished predictive evaluation from model explanation. Nested farm-grouped cross-validation and leave-one-farm-out validation assessed predictive performance, with model selection confined to the training data. Separately, an XGBoost (3.0.5) regression model was used for exploratory TreeSHAP analysis to describe how a specified tree-based model used the biosecurity and age-group variables.

Tree-based methods can represent non-linear relationships and interactions that may not be apparent in pairwise correlations or additive linear models [27,30,31]. However, their capacity to represent these relationships was not interpreted as evidence that a particular biological interaction existed. Explainability outputs described the fitted models and were considered alongside the continuous outcome associations and predictive validation results.

#### 2.10.1. Role of Exploratory Tree-Based Models

The initial exploratory workflow included a Random Forest classifier using the median-defined High Usage Class endpoint. Ensemble tree methods were considered suitable for exploring non-linear predictor relationships in the observational data [35]. However, internal classifier diagnostics were not used to substantiate predictive performance in unseen farms or the primary biosecurity–AMU associations.

The revised explainability analysis used XGBoost regression with the continuous log-transformed AMU index as its target. This preserved the quantitative outcome information and aligned the explanations with the regression-based predictive analysis. The continuous inferential models, secondary median-split Firth analysis and exploratory tree model explanations retained their distinct analytical roles. The reported SHAP results refer specifically to the XGBoost regression described below, rather than to the preliminary classifier.

#### 2.10.2. Regression-First Nested Grouped-CV Machine Learning Analysis

The regression-based machine learning analysis evaluated whether age-group and biosecurity information supported prediction of the log-transformed AMU index in farms not represented during training. Candidate models were Elastic Net, Random Forest Regressor, Extra Trees Regressor and XGBoost Regressor [35,36,37,38]. The analysis used compact hyperparameter search grids and a random seed of 42; the grids and selected settings are provided in the Supplementary Materials. The hyperparameter search grids and initial estimator configurations for Elastic Net, Random Forest, Extra Trees and XGBoost are provided in Supplementary Table S6.

Three feature sets were evaluated: age group alone; age group plus the nine recorded biosecurity variables; and age group plus the nine biosecurity variables, calendar year and calendar month. Farm identifier was used for grouping and was not included as a predictor. These feature sets were evaluated in both the unfiltered and filtered cohorts.

The outer validation loop used five farm-grouped folds. All observations from a farm were assigned to either the training partition or the held-out partition, ensuring that no farm appeared in both. Hyperparameter tuning within each outer training partition used three inner farm-grouped folds. The same inner tuning procedure was used in leave-one-farm-out validation, in which each farm was held out in turn. Neither validation scheme used shuffled observation-level splitting.

All candidate estimators were embedded in preprocessing pipelines. Age group was one-hot encoded within each training partition, with previously unseen categories handled without fitting to the held-out data. Numeric predictors were standardised using training partition parameters. All retained inputs were complete, so no imputation was required. Numeric age-group codes were excluded to avoid duplicating production-stage information. No PCA or factor scores derived from the full cohort entered the validation feature sets.

Hyperparameters were selected by mean inner-fold root mean squared error (RMSE), using farm-grouped grid search for all candidate families, including Elastic Net. Each tuned family was evaluated separately. An additional combined selection procedure chose both the model family and its hyperparameters using only the inner validation results within each outer training partition. The selected model was then fitted to that training partition and evaluated on its held-out farms. Individual-family results were retained as descriptive comparisons; outer held-out errors were not used to select a model and subsequently provide the same errors as an independent assessment of that selection.

Predictive performance was compared with a training-fold mean baseline, a training-fold stage-mean baseline and the age-group-only models. Baseline predictions were derived exclusively from training outcomes. These comparisons assessed whether biosecurity information provided predictive benefit beyond simpler representations of the outcome and production-stage structure.

For each procedure and validation scheme, pooled RMSE, mean absolute error (MAE) and coefficient of determination (R^2^) were calculated from the complete set of held-out predictions. Mean farm-level metrics were reported separately and were not treated as interchangeable with pooled metrics. Hyperparameter grids, fold assignments, selected settings, held-out predictions and performance summaries are provided in Supplementary Tables S6 and S7 and their associated outputs.

### 2.11. Explainability and Model Interpretation

Exploratory SHAP analysis was performed for the XGBoost regression using age group and the nine biosecurity variables. This model family was specified for explanation independently of the family selected by the combined predictive procedure. Its SHAP values therefore explain the corresponding XGBoost predictions, rather than predictions from another model family or a model chosen after comparing outer validation errors.

Within each of the five outer farm-grouped folds, XGBoost hyperparameters were tuned using the training farms. TreeSHAP values were then calculated for observations from the held-out farms, covering all 1110 unfiltered analytical units. Tree path-dependent expectations were based on the fitted trees’ training-node counts. Each fold model consequently had its own expected output, and the pooled SHAP summaries combined explanations from these fold-specific models.

Positive SHAP values indicated contributions that increased the model’s predicted log-transformed AMU index relative to its expected output; negative values indicated contributions that decreased it. Figure S1 presents the pooled SHAP beeswarm using the red/blue feature-value convention. For biosecurity variables, higher values, shown in red, represent poorer recorded conditions, while lower values, shown in blue, represent more favourable conditions according to Table S1. For one-hot age-group indicators, higher values indicate membership of the corresponding production stage. Feature-value colour and SHAP sign therefore convey different information.

Mean absolute SHAP contributions and farm-specific summaries were reported in the Supplementary Materials. Predictive reliance on the biosecurity variables was additionally examined by jointly permuting the nine-variable biosecurity block within held-out folds and evaluating the resulting change in prediction error. These permutation summaries were treated as model diagnostics rather than inferential tests of individual biosecurity effects.

SHAP and permutation results were interpreted with attention to correlated predictors, limited category support and farm-held-out predictive performance. They did not establish causal effects, externally validate the scoring instrument or independently demonstrate predictive utility. Agreement with the primary inferential results was considered descriptive consistency within the same dataset, rather than independent confirmation. Patterns confined to the exploratory models were treated as model-specific and hypothesis-generating.

### 2.12. Software and Reproducibility

All analyses were implemented using reproducible custom workflows in Python (Python Software Foundation, Wilmington, DE, USA) and R (R Foundation for Statistical Computing, Vienna, Austria). Data processing, aggregation, descriptive statistics, inferential modelling, machine learning analyses, and figure generation were performed primarily in Python. Data manipulation relied on pandas and numpy; inferential models were fitted using statsmodels; machine learning workflows used scikit-learn; SHAP-based explainability was performed with shap where supported; and boosted-tree models were fitted with xgboost when available. Firth bias-reduced logistic regression for the secondary binary sensitivity analyses was implemented separately in R 4.6.0 using the logistf package 1.26.1.

The nested-CV implementation audit recorded Python 3.11.9, scikit-learn 1.7.2, pandas 2.3.3, numpy 2.3.3, statsmodels 0.14.6, shap 0.48.0, xgboost 3.0.5, R 4.6.0, and logistf 1.26.1. The audit also documented the outer and inner grouped cross-validation splitters, parameter grids, scoring metric, feature sets, excluded predictors, fold-wise preprocessing steps, and leakage-control checks. In particular, the audit confirmed that farm identifiers were passed to both outer validation and inner tuning procedures, age group was represented only through fold-wise one-hot encoding, numeric age-group codes were excluded from manuscript-facing ML predictor sets, and precomputed PCA- or factor analysis-based hygiene scores were not used in the transportability audit. All outputs were written to scenario-specific directories to preserve the distinction between unfiltered and filtered cohorts, composite and separate-variable biosecurity specifications, and inferential, sensitivity, machine learning, and explainability branches. The workflow generated model coefficients, support diagnostics, grouped cross-validation summaries, Firth logistic outputs, bootstrap sensitivity results, influence diagnostics, figures, reproducibility logs, and run manifests. Software session information, random seeds, hyperparameter grids, fold assignments, feature set definitions, and leakage audit outputs were archived with the output files to support reproducibility.

### 2.13. Declaration of Generative AI and AI-Assisted Technologies in the Manuscript Preparation Process

During the preparation of this work, the authors used ChatGPT 5.5. in order to brainstorm research ideas and to correct grammatical errors in the manuscript. After using this tool, the authors reviewed and edited the content as needed and take full responsibility for the content of the published article.

## 3. Results

### 3.1. Cohort Construction, AMU Distribution, and Farm-Level Heterogeneity

**Figure 2:**
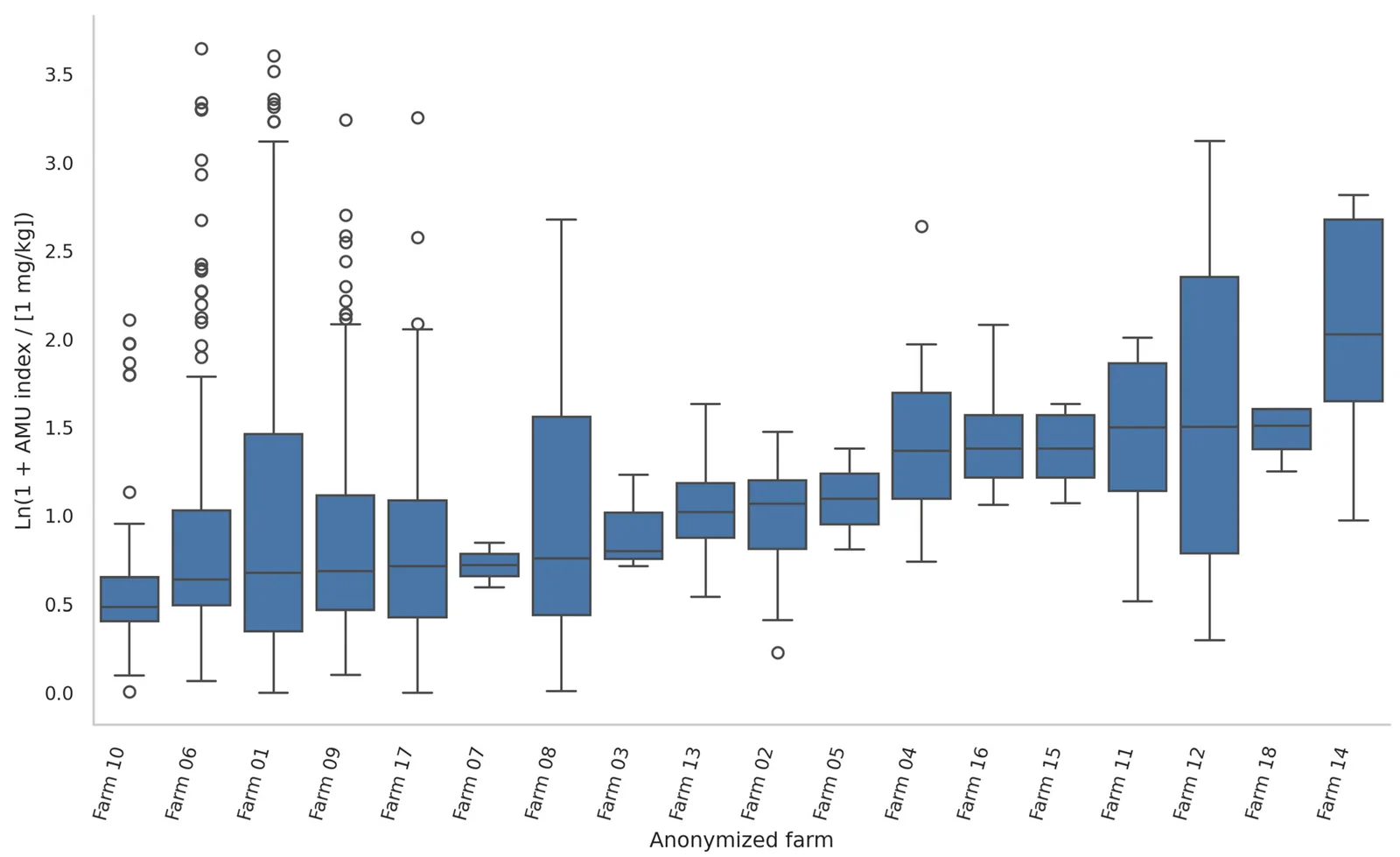
Boxplots of the log-transformed AMU index across anonymised farms in the unfiltered cohort, illustrating differences in recorded AMU and dispersion at the farm level.

The aggregated unfiltered analytical dataset contained 1110 farm–month–age-group batches from 18 farms (Figure 2). This unfiltered cohort was retained as the primary analytical population and therefore as the main basis for inference. A restricted filtered cohort was also evaluated as a sensitivity population. This filtered cohort retained 939 batches from 13 farms after excluding selected production-stage categories and records from five anonymised farms. Overall, filtering removed 171 aggregated batches, corresponding to 15.4% of the unfiltered dataset (Table 1). All retained analytical variables were complete, and these exclusions were unrelated to missing data.

The filtered cohort was developed during the analysis-development phase to examine whether model behaviour and biosecurity–AMU associations were sensitive to cohort heterogeneity, sparse subgroup support, and the inclusion of selected production-stage categories and farms. Because these restrictions changed both farm composition and age-group composition, the filtered cohort was not interpreted as a clean or inherently superior dataset. Instead, it was treated as a post hoc restricted population sensitivity cohort. Consequently, all primary interpretations were based on the unfiltered cohort, while filtered cohort results were used to assess robustness and support sensitivity.

Antimicrobial use, expressed using the sum-of-ratios AMU index and its logarithmic transformation defined in Section 2.2, showed substantial heterogeneity across farm–month–age-group observations. In the unfiltered cohort, the median untransformed AMU index was 1.121 mg/kg, with an interquartile range of 0.598–3.431 mg/kg. The empirical distribution of the log-transformed index remained broad and right-skewed, with an upper tail indicating markedly elevated recorded AMU in a subset of batches. All available AMU values were positive; use of a zero-compatible transformation did not indicate that zero-use observations were present.

**Figure 3:**
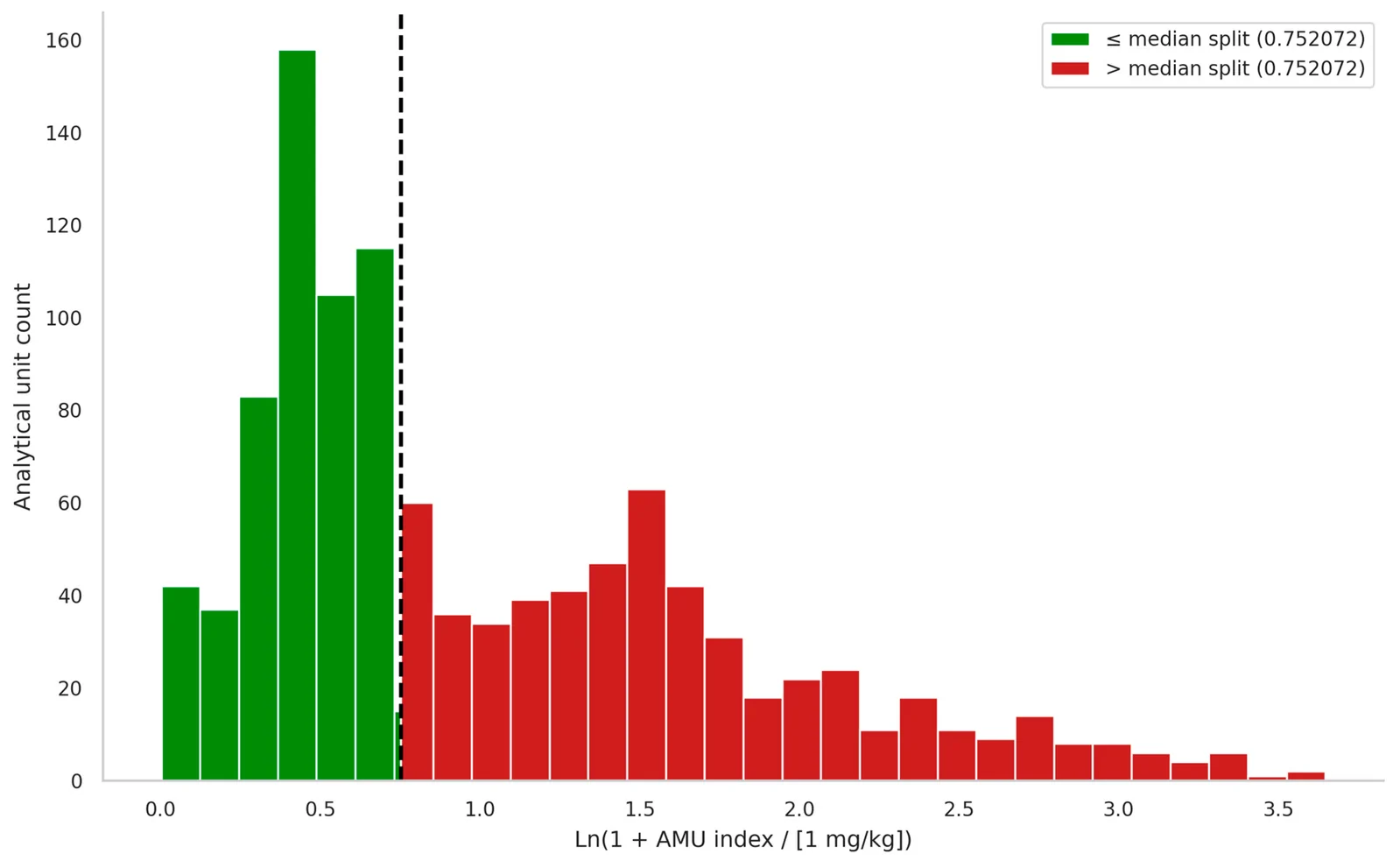
Distribution of the log-transformed AMU index across unfiltered farm–month–age-group batches, stratified by the cohort median of 0.752072054. The median split is descriptive and does not represent a clinical or regulatory threshold.

For secondary binary sensitivity analyses and descriptive visualisation, observations were divided at the cohort-specific median into lower- and higher-AMU groups. The log-transformed thresholds were 0.752072054 in the unfiltered cohort and 0.815935145 in the filtered cohort. Figure 3 shows the unfiltered distribution and its median split. This split was not used as the primary inferential endpoint; continuous log-transformed AMU remained the main outcome throughout the study.

Figure 3 showed that lower-AMU observations were concentrated within a narrower part of the log-AMU range, whereas higher-AMU observations displayed greater dispersion and included the most extreme values. This pattern described the wider upper portion of the observed distribution rather than establishing a distinct biological or management state.

Farm-level visualisations further emphasised the heterogeneous structure of AMU. Anonymised farm-level summaries showed that some farms contributed predominantly lower-AMU observations, others contributed mainly higher-AMU observations, and several farms showed mixed patterns across time and/or production stages. Within-farm time-series plots also indicated that mean recorded AMU varied over time rather than remaining constant. Dotted line shows the median. (Figure S2). These monthly summaries could also be influenced by the production stages represented in each farm month. This combination of between-farm and within-farm heterogeneity supported accounting for farm clustering in the inferential models and evaluating prediction with farm-held-out validation. Overall, recorded AMU varied across farm, calendar and production-stage contexts rather than behaving as a uniform farm-level trait.

### 3.2. Correlation Structure and Hygiene-Component Organization

Correlation analyses showed that pairwise relationships between the log-transformed AMU index and individual biosecurity variables were generally weak. Log-transformed AMU showed correlations close to zero with Animal Population Separation (r = −0.029) and the mean coded hygiene score (r = −0.005). Age group was excluded from the numerical correlation matrix because its categories do not have a meaningful numerical ordering.

**Figure 4:**
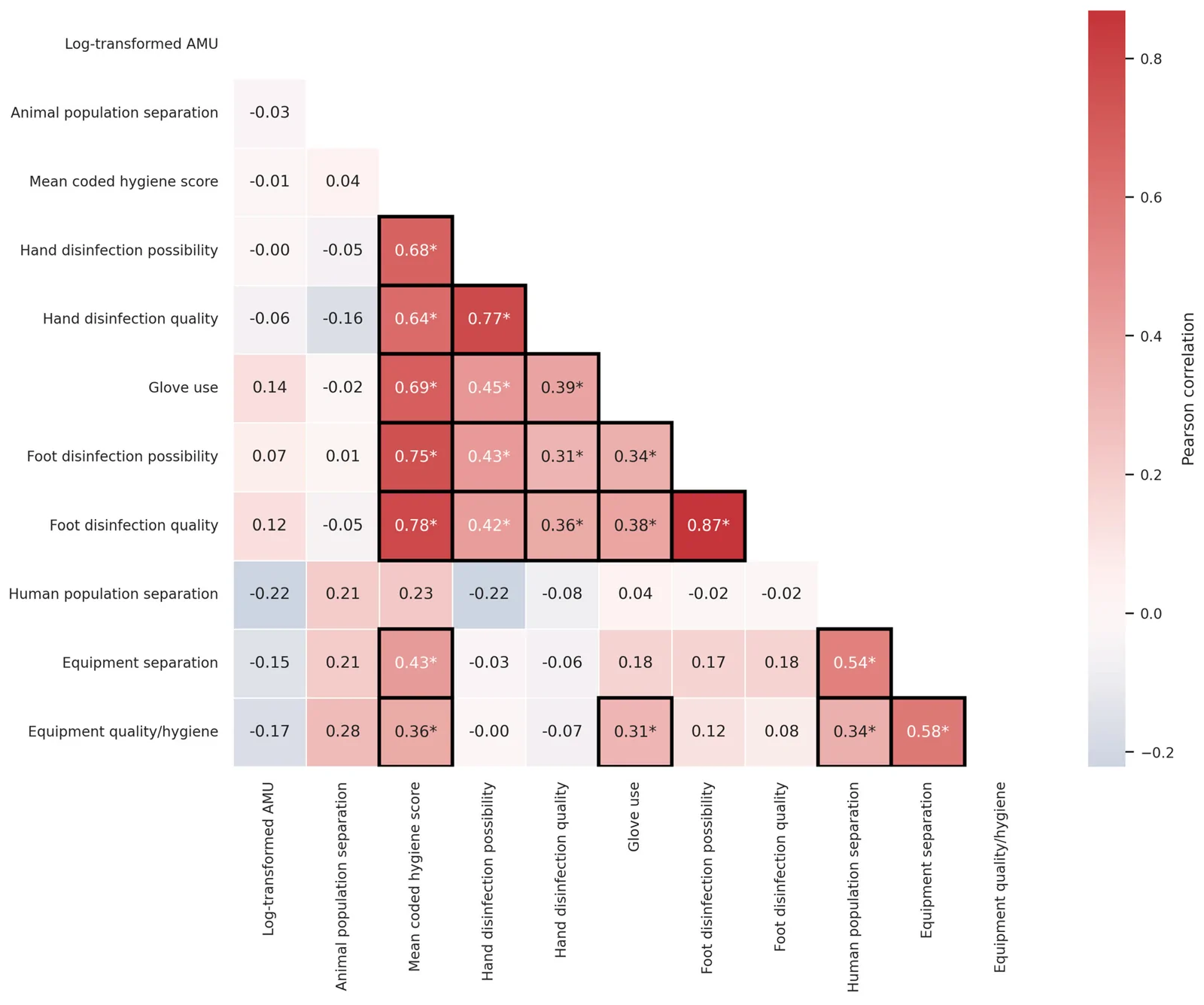
Pearson correlation matrix of the log-transformed AMU index, Animal Population Separation, the mean coded hygiene score and individual hygiene-related biosecurity variables. Numeric age-group codes are excluded. * Boxed cells indicate |r| ≥ 0.30, a descriptive display threshold rather than a statistical significance criterion. Correlations between the mean score and its components are partly mathematical.

Relationships among the biosecurity variables ranged from weak to strong. Strong positive correlations were observed between hand disinfection availability and practice (r = 0.775) and between foot disinfection availability and practice (r = 0.869). Moderate positive relationships were observed between glove use and hand disinfection availability or practice (r = 0.445 and 0.390, respectively), between hand and foot disinfection indicators (r = 0.314–0.431), and between glove use and foot disinfection indicators (r = 0.338–0.381). Personnel separation correlated with equipment separation (r = 0.538) and equipment condition and hygiene (r = 0.336), while equipment separation correlated with equipment condition and hygiene (r = 0.583). Most remaining pairwise correlations were weaker. The indicators therefore captured partly overlapping dimensions, with particularly substantial overlap between the availability and practice measures within each disinfection domain (Figure 4).

Simple AMU–biosecurity correlations were weaker than these within-biosecurity relationships. Log-transformed AMU showed weak positive correlations with glove use (r = 0.136), foot disinfection availability (r = 0.068) and foot disinfection practice (r = 0.125), and weak negative correlations with personnel separation (r = −0.221), equipment separation (r = −0.146) and equipment condition and hygiene (r = −0.166). Thus, the descriptive correlation structure did not identify a dominant single linear biosecurity predictor of AMU.

These correlations describe the numerical codes assigned to the recorded categories. Correlations between the mean coded hygiene score and its constituent variables are partly mathematical because those variables contribute directly to the mean. Spearman correlations are provided as a supplementary sensitivity summary.

To characterise the internal hygiene structure more directly, the eight hygiene-related biosecurity variables were examined using principal component analysis of standardised codes. The first two components explained 63.0% of the variance in the unfiltered cohort and 62.4% in the filtered cohort, with similar loading patterns across the two populations. In the unfiltered cohort, the first and second components explained 37.0% and 26.0%, respectively; the corresponding proportions in the filtered cohort were 36.6% and 25.9%.

The first component was primarily defined by direct hygiene measures, including hand disinfection, foot disinfection and glove use. The second component was more strongly associated with personnel separation, equipment separation, and equipment condition and hygiene. These results described a similar low-dimensional organisation in the two overlapping cohorts, despite the weak pairwise associations between the hygiene variables and AMU. PCA loading signs are arbitrary and were oriented consistently for display.

**Figure 5:**
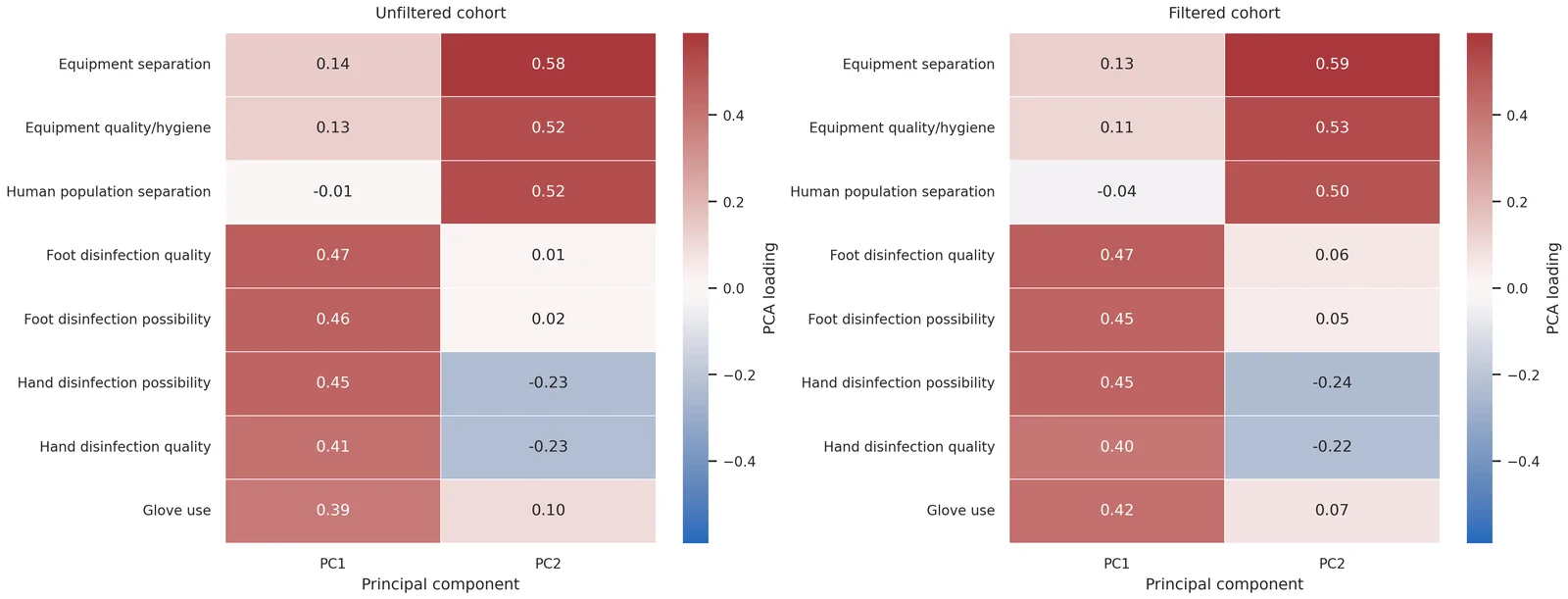
Principal component loading heatmaps for the eight hygiene-related biosecurity variables in the unfiltered and filtered cohorts. PCA was performed on standardised numeric codes. Component signs were oriented for display; the loading structure is descriptive and does not validate the scoring instrument.

The correlation and PCA results provided descriptive context for evaluating production stage, animal separation and broader hygiene-management conditions jointly in multivariable models (Figure 5 and Figures S3 and S4). They did not establish external validity of the scoring instrument or demonstrate that the components represented independent biological mechanisms.

### 3.3. Primary Inferential Analysis of Continuous AMU Across the 2 × 2 Scenario Matrix

The primary inferential endpoint was continuous log-transformed AMU. Four analytical scenarios were examined in a 2 × 2 matrix defined by cohort type, unfiltered versus filtered, and biosecurity representation, composite hygiene index versus separate biosecurity variables. Linear mixed-effects models with farm-level random intercepts were initially specified, but the random-effect variance collapsed in the final runs. Therefore, ordinary least squares models with farm cluster robust standard errors were retained as the primary continuous outcome estimator. Across the 2 × 2 scenario matrix, Animal Population Separation showed a positive but non-monotonic association with log-transformed AMU. The clearest and most reproducible continuous model signal was observed for Animal Population Separation code 2 in the separate-variable specification. In the primary unfiltered separate-variable model, code 2 was associated with higher log-AMU compared with code 0, the reference category (β = 0.205; 95% CI: 0.027 to 0.384; *p* = 0.024). In the filtered separate-variable sensitivity model, the corresponding association was stronger and more precisely estimated (β = 0.272; 95% CI: 0.140 to 0.405; *p* < 0.001). Animal Population Separation code 3 was also positive in direction, but its support was less stable. In the unfiltered separate-variable model, the code 3 estimate was positive but imprecise (β = 0.337; 95% CI: −0.122 to 0.795; *p* = 0.150). In the filtered separate-variable model, the corresponding estimate remained positive but was again uncertain (β = 0.264; 95% CI: −0.217 to 0.745; *p* = 0.282). Thus, although the highest separation category pointed toward increased AMU, the continuous models did not support a simple monotonic dose–response across all ordinal codes.

Composite hygiene index models showed a broadly similar directional pattern but weaker or more specification-dependent support. In the unfiltered composite model using the mean hygiene index, Animal Population Separation code 3 was positive but did not reach conventional statistical significance (β = 0.343; 95% CI: −0.053 to 0.738; *p* = 0.090), while code 2 showed little evidence of association (β = 0.045; 95% CI: −0.135 to 0.225; *p* = 0.622). In the filtered composite model using the mean hygiene index, code 3 again showed a positive but uncertain association (β = 0.374; 95% CI: −0.049 to 0.797; *p* = 0.083), whereas code 2 remained weaker (β = 0.112; 95% CI: −0.054 to 0.279; *p* = 0.187). Sensitivity composite variants based on PCA, factor analysis, and weighted hygiene indices yielded comparable directional patterns, with some strengthening of code 2 support in the filtered factor index model (Table 2).

**Figure 6:**
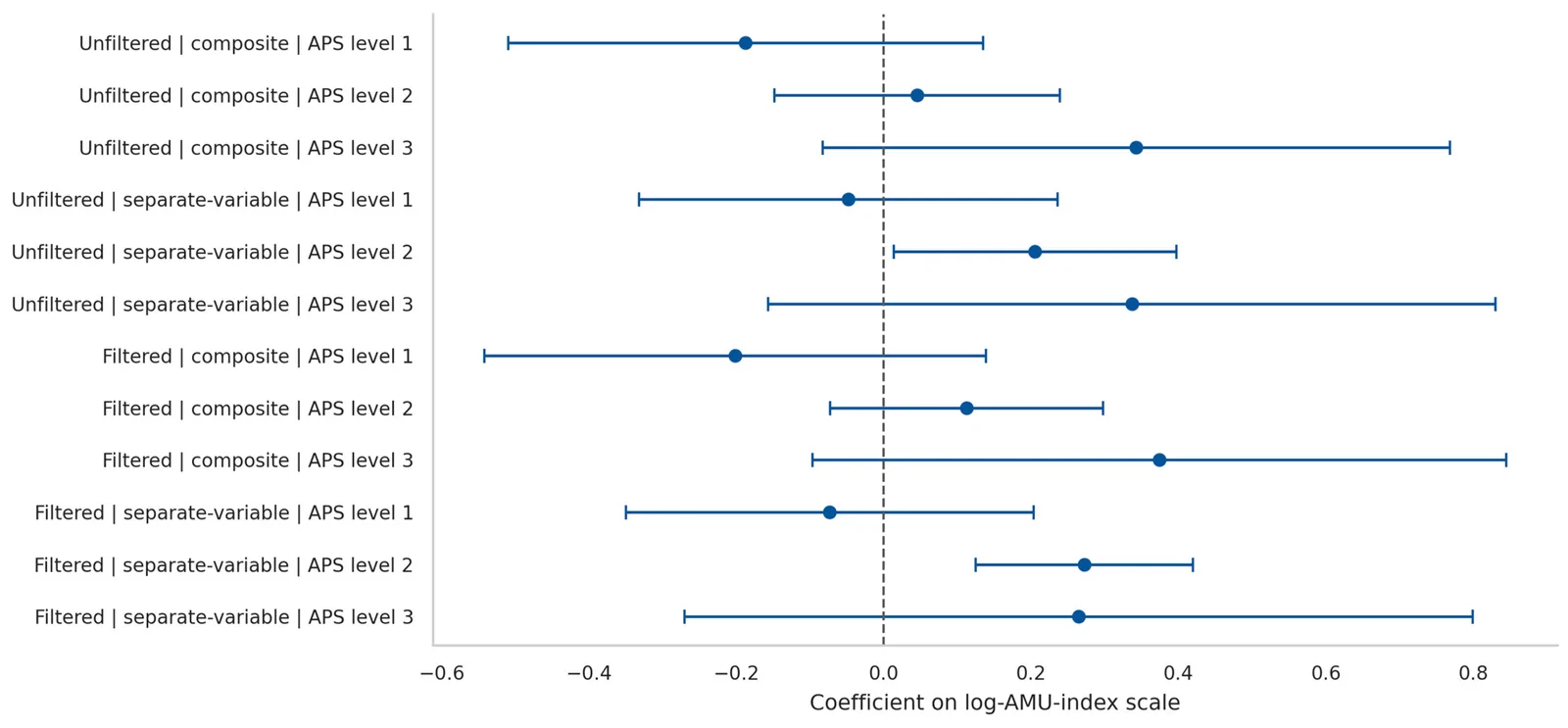
Forest plot of Animal Population Separation coefficients across the four unfiltered/filtered and composite/separate-variable continuous outcome models. The 12 entries represent codes 1–3 versus code 0 in each specification; intervals are CR1-t 95% confidence intervals. Code 0 denotes strict all-in/all-out management; code 1 denotes movement between age groups involving <5% of animals; code 2 denotes movement within age groups; and code 3 denotes mixing between groups that is continuous or possible at any time, with support and farm context.

Taken together, the continuous outcome models indicate that impaired Animal Population Separation is associated with higher AMU, but the pattern is not adequately described as a linear ordinal gradient (Figure 6). In the primary models, the clearest adjusted association appeared at separation level 2 in the separate-variable specification, whereas level 3 remained directionally adverse but more unstable across specifications. This suggests that the ordinal separation categories may represent heterogeneous management states rather than evenly spaced increments of biosecurity impairment. The stronger statistical support for code 2 should therefore not be interpreted as evidence that code 2 is biologically more severe than code 3. Rather, code 2 provided a more stable contrast against the reference category, whereas code 3 was sparsely represented and more strongly influenced by farm-level composition. The results are therefore best interpreted as evidence that impaired Animal Population Separation is associated with higher AMU, while the specific ordinal contrast with the strongest statistical support depends on the data. The primary inferential endpoint was the continuous log-transformed AMU index. Four analytical scenarios were examined in a 2 × 2 matrix defined by cohort type, unfiltered versus filtered, and biosecurity representation, mean coded hygiene score versus separate biosecurity variables. Ordinary least squares models with farm-clustered CR1 covariance and t-based uncertainty were used as the primary continuous outcome estimator.

Animal Population Separation codes were interpreted using Supplementary Table S1. Code 0, the reference category, denoted consistently implemented strict all-in/all-out management; code 1 denoted movement between age groups involving <5% of animals; code 2 denoted movement within age groups; and code 3 denoted mixing between animal groups that was continuous or possible at any time.

Across the 2 × 2 scenario matrix, estimates for separation codes 2 and 3 were positive, whereas estimates for code 1 were negative. The pattern therefore did not follow a monotonic gradient. The clearest conventional coefficient-level evidence appeared for code 2 in the separate-variable specification. In the primary unfiltered separate-variable model, code 2 was associated with higher log-transformed AMU compared with code 0 (β = 0.205; CR1-t 95% CI 0.013–0.397; *p* = 0.037). In the filtered separate-variable sensitivity model, the corresponding estimate was larger and its conventional interval narrower (β = 0.272; 95% CI 0.125–0.419; *p* = 0.002).

Separation code 3 was also positive in direction but less precisely estimated. In the unfiltered separate-variable model, its coefficient was 0.337 (95% CI −0.157–0.830; *p* = 0.168). In the filtered separate-variable model, the corresponding estimate was 0.264 (95% CI −0.270–0.799; *p* = 0.303). The continuous models therefore did not establish an increasing AMU gradient across all separation categories.

Composite models showed a broadly similar directional pattern but weaker conventional statistical support. In the unfiltered model using the mean coded hygiene score, the code 3 estimate was 0.343 (95% CI −0.083–0.768; *p* = 0.108), while the code 2 estimate was 0.045 (95% CI −0.149–0.239; *p* = 0.629). In the filtered composite model, the code 3 estimate was 0.374 (95% CI −0.096–0.845; *p* = 0.109), and the code 2 estimate was 0.112 (95% CI −0.073–0.298; *p* = 0.211).

Sensitivity composite variants based on PCA, one-factor analysis and weighted averaging yielded comparable directional patterns (Table S8). The filtered one-factor model showed a somewhat larger code 2 estimate than the mean-score model, but its CR1-t interval included zero (β = 0.139; 95% CI −0.002–0.280; *p* = 0.053).

All models adjust for categorical age group. Composite models additionally include the mean coded hygiene score; separate-variable models include the eight individual numeric hygiene covariates. Lower separation codes denote more favourable recorded conditions, with code 0 representing strict all-in/all-out management. Category definitions are provided in Supplementary Table S1. Conventional *p*-values should be considered alongside the wild-cluster tests in Supplementary Table S9.

Wild-cluster inference qualified the conventional code 2 findings. In the unfiltered separate-variable model, the code 2 wild-cluster *p*-value was 0.116, compared with 0.040 in the filtered model. The corresponding joint tests of all separation contrasts yielded *p*-values of 0.394 and 0.098, respectively. Conventional significance in selected contrasts therefore did not establish a robust overall separation–AMU association. Additional calendar, categorical hygiene and farm intercept specifications showed that estimates depended on adjustment and biosecurity representation (Table S10). Leave-one-farm-out influence diagnostics are shown in Figure S5.

Taken together, the continuous outcome models identified positive code 2 and code 3 contrasts whose statistical support depended on specification, cohort composition and uncertainty estimation (Figure 6). Stronger conventional support for code 2 should not be interpreted as evidence that this category is biologically more severe than code 3. Code 2 had broader support, whereas code 3 was sparsely represented and concentrated within a small number of farms. The results therefore describe model-dependent associations between recorded separation conditions and AMU.

The stage-by-separation support audit did not meet the stated screen for a full interaction analysis. Only the suckling piglet stratum supported the stage-specific analysis under the screening and design-rank criteria, comprising 187 units from 12 farms. Its three separation contrasts had wide confidence intervals that included zero (Table S5). These results did not establish whether the separation–AMU association differed across production stages.

### 3.4. Secondary Binary Sensitivity Analyses

Binary high-AMU models were fitted as secondary sensitivity analyses only. The endpoint was defined using the median log-transformed AMU index within each cohort. In the unfiltered cohort, this produced 555 high-AMU and 555 low-AMU observations; in the filtered cohort, 469 high-AMU and 470 low-AMU observations were available.

Cohort-specific support diagnostics showed sparse outcome support in the highest Animal Population Separation category. In the filtered cohort, all 25 observations in code 3 were classified as high-AMU, with no low-AMU observations. These units came from three farms, and 22 were contributed by one farm. In the unfiltered cohort, code 3 contained 31 high-AMU and two low-AMU observations among 33 units from seven farms, again with 22 units from one farm. These patterns presented complete or near-complete outcome separation and justified using Firth penalised estimation for the secondary binary contrasts.

The Firth point estimates indicated elevated odds of high AMU for codes 2 and 3 relative to code 0. In the unfiltered composite model, the odds ratios were 2.697 for code 2 and 26.194 for code 3. In the unfiltered separate-variable model, the corresponding odds ratios were 5.088 and 60.744. Farm bootstrap confidence intervals were withheld for these models because only 1097 and 1123 of the 1999 requested draws, respectively, were estimable, below the 90% reporting criterion.

Filtered cohort Firth models showed the same direction for codes 2 and 3. In the filtered composite model, the code 2 odds ratio was 4.897, with an exploratory farm bootstrap 95% interval of 1.646–28.296; the code 3 odds ratio was 64.617, with an interval of 11.466–439.883. In the filtered separate-variable model, the corresponding estimates were 9.362 for code 2 (95% interval 2.522–32.697) and 42.035 for code 3 (95% interval 2.573–888.832). These models had 1836 and 1838 estimable bootstrap draws, respectively. The particularly wide code 3 intervals reflected the limited information available for that category and did not permit precise effect quantification.

Overall, the binary point estimates were consistent with higher odds of above-median AMU in separation codes 2 and 3, but sparse support, farm concentration and bootstrap instability limited their interpretation. They were retained as secondary sensitivity evidence rather than causal risk estimates or confirmation of a uniformly robust association. The continuous outcome models remained the primary basis for inference. Binary support, point estimates and bootstrap diagnostics are reported in Table S3: Farm-held-out machine learning transportability.

A machine learning transportability audit was performed using the continuous log-transformed AMU index as the target. The purpose was to evaluate prediction in unseen farms rather than internal fit within farms represented during training. Validation used nested farm-grouped cross-validation, with farm identifiers defining both the outer validation groups and the inner tuning groups. Scaling and one-hot encoding were fitted within the corresponding training partitions; no imputation was required because the retained predictors were complete.

Age group was represented categorically through training partition one-hot encoding, and numeric age-group codes were excluded. Precomputed PCA and factor scores were also excluded to avoid leakage from full-dataset composite derivation. Feature sets comprised age group alone, biosecurity plus age group, and biosecurity plus age group and calendar variables.

Under nested grouped validation in the unfiltered cohort, the procedure that selected model family and hyperparameters within training folds achieved pooled RMSE 0.780 and R^2^ −0.101 using biosecurity plus age group. This compared with RMSE 0.765 for the corresponding age-group-only procedure, 0.763 for the training-fold mean baseline and 0.743 for the training-fold stage-mean baseline. Adding calendar variables yielded RMSE 0.793. In the filtered cohort, the corresponding biosecurity-plus-age RMSE was 0.803, compared with 0.786 for age group alone, 0.771 for the mean baseline and 0.766 for the stage-mean baseline (Table 3).

**Figure 7:**
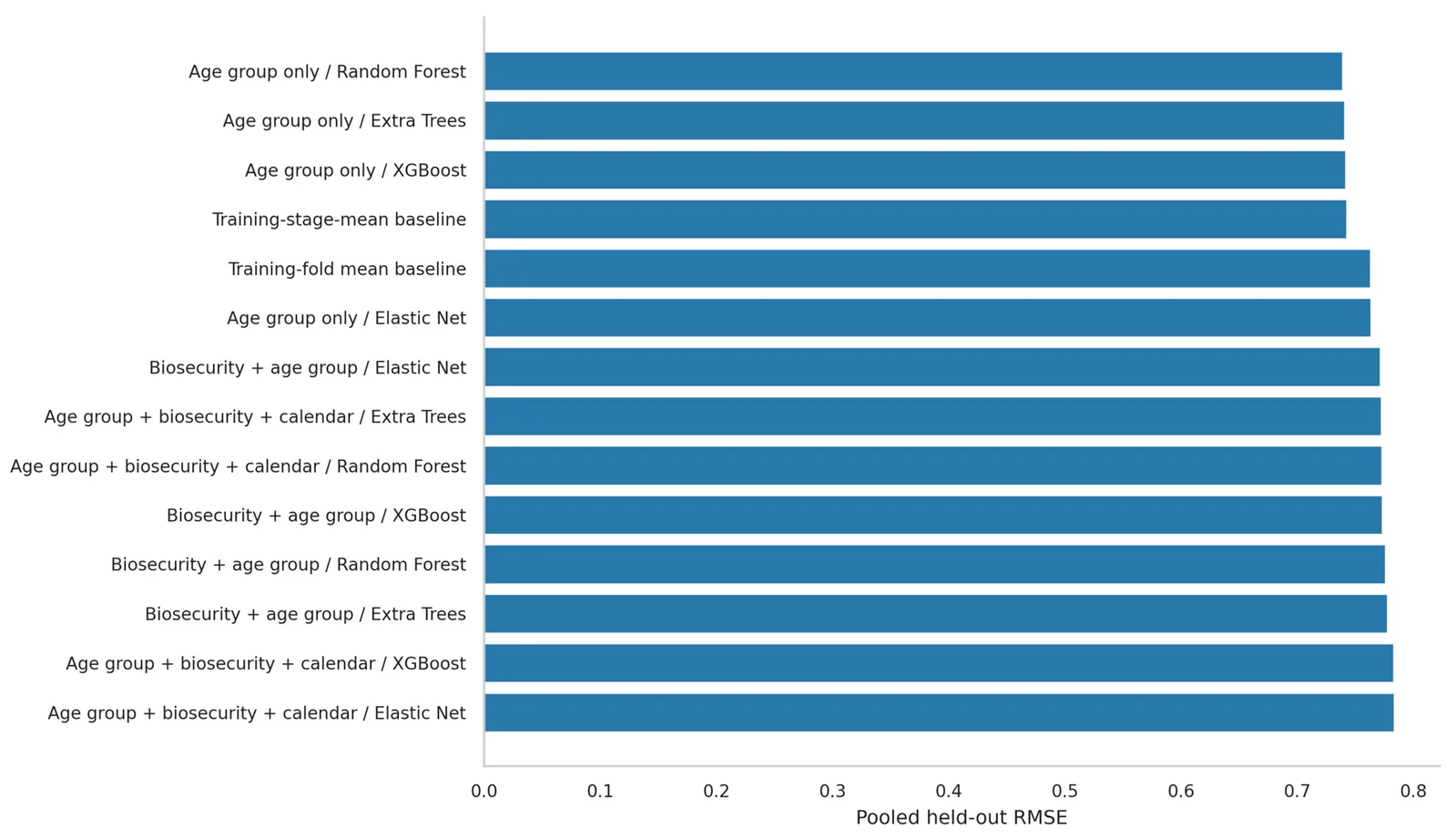
Pooled held-out RMSE comparisons for prediction of the continuous log-transformed AMU index using nested farm-grouped cross-validation in the unfiltered cohort. Each family was tuned within training farms. Individual-family comparisons are descriptive; model-family selection for the combined procedure occurred within the inner validation loop.

Individual-family comparisons were retained descriptively in Figure 7 and the supplementary results. Among the individual tuned families, the lowest unfiltered nested-validation RMSE was obtained by Random Forest using age group alone (RMSE 0.739; pooled R^2^ 0.011). In the filtered cohort, XGBoost using age group alone had the lowest corresponding RMSE (0.766; pooled R^2^ −0.048). These comparisons were not used to select a family after inspecting the outer results and then claim independently validated performance for that choice.

Leave-one-farm-out validation showed a similar lack of consistent incremental benefit from biosecurity. In the unfiltered cohort, the inner-selected biosecurity-plus-age procedure achieved RMSE 0.810 and pooled R^2^ −0.187, compared with RMSE 0.770 for age group alone, 0.759 for the mean baseline and 0.742 for the stage-mean baseline. In the filtered cohort, biosecurity plus age group achieved RMSE 0.782, modestly lower than the inner-selected age-only procedure at 0.789, but higher than the mean and stage-mean baselines at 0.766 and 0.764. Adding calendar variables yielded RMSE 0.782 after rounding.

**Figure 8:**
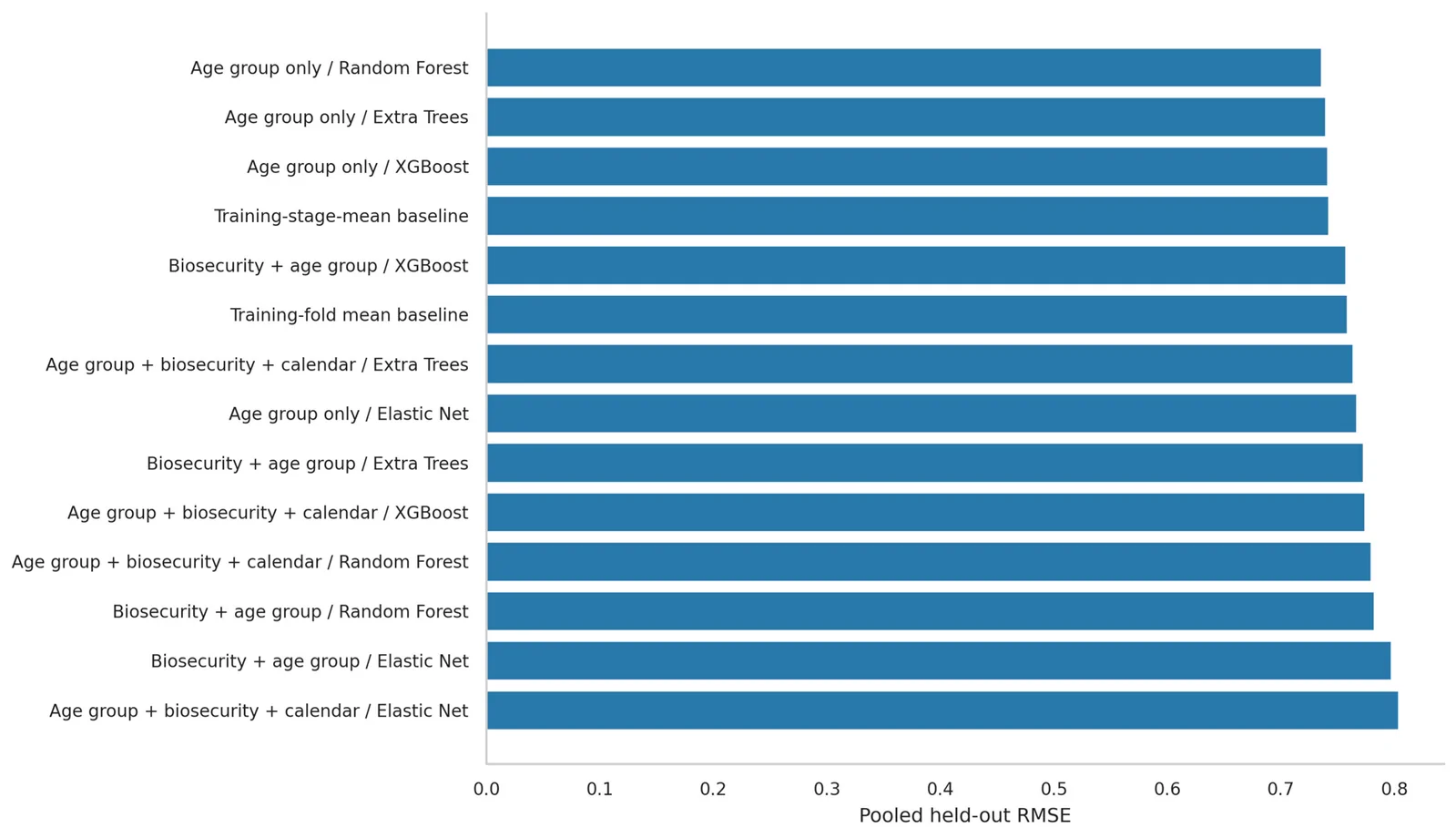
Pooled leave-one-farm-out prediction errors for the continuous log-transformed AMU index in the unfiltered cohort. Hyperparameter tuning used the remaining training farms, and baseline predictions were derived exclusively from training outcomes.

The individual-family leave-one-farm-out comparisons likewise favoured simple production-stage information: Random Forest using age group alone had the lowest unfiltered pooled RMSE of 0.736, while XGBoost using age group alone had the lowest filtered pooled RMSE of 0.764. These remain descriptive comparisons among the evaluated families (Figure 8 and Figure S6).

Model family and hyperparameters were selected separately within the training partitions for each feature set. Baseline predictions used training outcomes only. Individual-family comparisons and mean farm-level metrics are reported separately in Supplementary Table S7 and associated outputs. Small differences may be obscured by rounding.

Overall, the available biosecurity variables did not establish consistent predictive improvement in unseen farms beyond production-stage information and simple baselines. The modest improvement over the inner-selected age-only procedure in filtered leave-one-farm-out validation did not extend to the simpler baselines. Pooled R^2^ and mean farm-level R^2^ were distinguished throughout because they summarise performance differently and are not interchangeable. The machine learning branch was therefore interpreted as a transportability assessment and exploratory model behaviour layer rather than evidence of a deployment-ready AMU prediction model.

### 3.5. Exploratory Explainability of Fitted Tree-Based Models

Exploratory machine learning analyses examined whether multivariable biosecurity and age-group information contained patterns beyond simple pairwise correlations. In the archived preliminary analysis of the filtered cohort, a Random Forest classifier using the median-based High Usage Class endpoint achieved accuracy 0.7500 and ROC AUC 0.8537 in its internal evaluation. These historical classifier diagnostics were retained as exploratory context; they were not results from the revised farm-held-out regression analysis and were not interpreted as evidence of prediction in unseen farms.

The revised SHAP analysis used XGBoost regression with biosecurity and age group to explain predictions for held-out farms. It covered all 1110 unfiltered analytical units across five outer folds. The corresponding pooled SHAP summary is provided in Figure S1.

The largest pooled mean absolute SHAP contributions included the post-weaning production-stage indicator, labelled “Post-nursery pig” in the figures (0.114), the fattening-stage indicator (0.089), personnel separation (0.075) and foot disinfection availability (0.050). Other hygiene variables and Animal Population Separation also contributed to fitted predictions; the mean absolute contribution for Animal Population Separation was 0.023. Full feature-level and farm-specific summaries are provided in Table S11 and its associated outputs.

These rankings describe how the fold-specific XGBoost models used the available predictors. They do not rank causal or biological importance. The pooled beeswarm combines explanations from different training-fold models, each with its own expected output. Positive SHAP values increase the corresponding model’s prediction relative to that expected output. For the biosecurity variables, red indicates higher codes and poorer recorded conditions, while blue indicates lower codes and more favourable conditions; age-group indicator colours instead distinguish stage membership.

Held-out biosecurity-block permutation diagnostics and supplementary SHAP summaries provided additional descriptions of model reliance on the recorded predictors. Their interpretation was constrained by correlations among the variables, limited category support and weak predictive improvement from biosecurity in the farm-held-out comparisons. The explainability outputs therefore did not establish a validated cross-farm predictor.

Overall, the explainable ML branch described multivariable structure in the fitted models but did not provide independent evidence that biosecurity variables could predict AMU reliably in unseen farms. The primary interpretation remained based on the continuous outcome inferential models and their sensitivity analyses.

## 4. Discussion

This study examined the relationship between farm-level biosecurity practices and antimicrobial use (AMU) in pig production using an integrated epidemiological and explainable ML framework. Four main findings emerge from the analyses. First, AMU showed substantial heterogeneity across farms and farm–month–age-group batches, supporting its interpretation as a context-dependent management outcome rather than a uniform farm trait. Second, simple pairwise associations between AMU and individual biosecurity variables were generally weak, while multivariable analyses described relationships involving production stage, hygiene-related conditions and Animal Population Separation. Third, recorded separation contrasts showed model-dependent associations with the AMU index. Conventional statistical support was strongest for separation code 2 in the separate-variable specification, but this finding was sensitive to hygiene adjustment and the method used to estimate uncertainty. The highest separation category remained positive in direction but was sparsely supported and imprecisely estimated. Fourth, farm-held-out machine learning validation showed limited predictive benefit from the available biosecurity variables beyond production-stage information and simple baselines.

Age group was an important structural covariate across the analytical branches. This is biologically plausible and aligns with established production knowledge, since antimicrobial treatments are often concentrated during early-life stages and around weaning, when piglets encounter multiple stressors, immature immune function and increased susceptibility to enteric and systemic disease [6,8]. Age-group adjustment was therefore important when examining biosecurity–AMU associations in populations containing different production stages. However, adjustment did not establish that residual associations were independent of all stage-related differences in disease pressure or management. Moreover, sparse stage-by-separation support prevented a reliable comparison of the separation–AMU association across production stages. The available suckling piglet analysis was inconclusive and did not establish the absence of effect modification.

Animal Population Separation provided an operationally interpretable exposure because the confirmed codebook links its categories to specific management conditions. Code 0 denotes consistently implemented strict all-in/all-out management, code 2 denotes animal movement within age groups, and code 3 denotes mixing between groups that is continuous or possible at any time. Although its simple correlation with AMU was weak, the multivariable continuous outcome models yielded positive code 2 and code 3 contrasts after adjustment for age group and hygiene-related covariates. The strongest conventional coefficient-level support occurred for code 2 in the separate-variable specification, while the highest category remained more imprecisely estimated.

These findings do not establish a monotonic dose–response or a biological threshold. The categories represent distinct management conditions, and their numerical ordering does not imply equally spaced biological effects. In particular, stronger statistical support for code 2 does not establish that movement within age groups is biologically more adverse than the conditions represented by code 3.

The biological rationale for investigating animal separation nevertheless remains relevant. Internal biosecurity in pig production aims to limit within-farm pathogen transmission through separation of age groups, avoidance of commingling, appropriate working lines and all-in/all-out production flow [39]. FAO good practice guidance similarly emphasises age-segregated rearing, avoidance of mixing pigs of different health status and all-in/all-out systems as measures intended to limit transmission and facilitate sanitation [40]. Previous pig herd studies have reported associations between internal biosecurity, farm management practices, treatment patterns and AMU levels [15,20]. These mechanisms provide plausible explanations for why separation conditions might be associated with AMU. The present observational analysis, however, did not directly measure pathogen transmission or estimate the causal effect of changing a separation practice.

The role of hygiene was more nuanced. Hygiene-related variables contributed to both the inferential and machine learning analyses, but their associations varied with representation and adjustment. Principal component analysis described a similar two-component organisation in the unfiltered and filtered cohorts. The first component primarily represented direct hygiene measures, including hand disinfection, foot disinfection and glove use, whereas the second reflected personnel separation and equipment management dimensions. Together, the two components accounted for approximately 63% of the variance in the standardised hygiene codes.

This similarity across overlapping cohorts suggested a consistent descriptive organisation of the recorded variables, but it did not validate a latent biosecurity construct or demonstrate measurement equivalence between farms. Strong correlations between disinfection availability and practice also showed that some indicators contained substantially overlapping information. Mixed directions and uncertainty in individual hygiene coefficients may therefore reflect correlated predictors, category support, adjustment choices and unmeasured management context, as well as possible differences between practices. The interpretation of hygiene as part of a broader management environment is compatible with previous evidence that biosecurity and management measures operate as interrelated farm-level systems rather than isolated interventions [16,19,20], but the present coefficient patterns do not identify how those systems act causally.

The filtering analysis provided an important methodological caution. The filtered cohort excluded 171 of 1110 aggregated batches, corresponding to 15.4% of the data, and reduced the number of farms from 18 to 13. Because filtering changed both farm and age-group composition, it was interpreted as a restricted-population sensitivity analysis rather than neutral data cleaning. The restriction was developed after data inspection and model development, so a stronger association in the filtered cohort could not be treated as evidence that restriction had recovered a less biased estimate. Filtered results were useful for examining sensitivity to cohort composition and exposure support, while the unfiltered cohort remained the main inferential population.

The uncertainty and influence analyses further qualified the separation findings. In the unfiltered separate-variable model, the code 2 coefficient had a conventional CR1-t *p*-value of 0.037 but a wild-cluster *p*-value of 0.116. After restriction, the corresponding values were 0.002 and 0.040. The joint wild-cluster tests of all separation contrasts yielded *p*-values of 0.394 and 0.098 in the unfiltered and filtered separate-variable models, respectively. Thus, the primary code 2 finding was sensitive to the uncertainty procedure, and the joint tests did not provide conventional statistical support for an overall separation association. These results do not prove that an association is absent; they indicate that the available data do not support describing it as consistently robust.

The contrast between code 2 and code 3 also needs to be considered in relation to farm support. Code 2 had broader representation, whereas code 3 contained only 33 units from seven farms in the unfiltered cohort and 25 units from three farms after restriction. In both cohorts, 22 code 3 units came from one farm. Leave-one-farm-out diagnostics, alternative hygiene representations, calendar adjustment and farm intercept sensitivities therefore informed interpretation of the estimates. The strongest conventional contrast depended on data support, adjustment and farm composition rather than establishing a universal ordering of management effects.

The binary high-AMU analyses showed broadly similar point-estimate directions but did not provide independent confirmation of the continuous outcome findings. The endpoint was derived by dichotomising the same AMU index at its cohort-specific median, and some separation strata had sparse or completely separated outcomes. Firth penalisation produced finite point estimates but did not create additional farm-level information, or itself address within-farm dependence. Farm bootstrap intervals were withheld in the unfiltered models because the proportion of estimable draws fell below the reporting criterion. The filtered models produced wide exploratory intervals, particularly for code 3. These analyses were therefore retained as secondary sensitivity results and did not permit precise risk quantification.

The machine learning results clarified the distinction between internal model behaviour and prediction in unseen farms. The preliminary Random Forest classifier achieved relatively good discrimination in its archived internal evaluation, but it used a median-dichotomised endpoint and did not establish predictive transportability to farms excluded from training. In the regression-based analysis, nested grouped cross-validation and leave-one-farm-out validation assessed prediction of the continuous log-transformed AMU index using farm-separated training and evaluation partitions.

Biosecurity-inclusive procedures did not consistently improve on age-group information or simple baselines. In the filtered leave-one-farm-out analysis, the inner-selected biosecurity-inclusive procedures modestly improved on the corresponding inner-selected age-only procedure, but remained worse than the training-fold mean and stage-mean baselines. Individual-family comparisons sometimes favoured age-group-only models, with only small differences from simpler predictions. These descriptive comparisons were distinguished from the procedure that selected model family within training folds, avoiding selection of a purportedly validated winner using the same outer errors subsequently reported as its performance.

These findings define the evidential role of the explainability component. Held-out SHAP summaries described how the specified XGBoost models used production-stage and biosecurity information. Prominent contributions included the post-weaning and fattening indicators, personnel separation and foot disinfection availability. Animal Population Separation and the other hygiene variables also contributed to predictions, but their SHAP magnitudes did not establish causal or biological importance. The summaries combined explanations from different fold models, and correlated predictors could share or redistribute contributions.

Given the limited predictive benefit observed in farm-held-out validation, SHAP and block-permutation outputs were interpreted as exploratory descriptions of model behaviour rather than evidence of a validated cross-farm prediction model. Additional information on disease pressure, treatment indications, veterinary decision-making, production flow and temporal changes may improve future modelling, but its incremental predictive value would need to be evaluated in independent farms.

Several limitations should be acknowledged. First, the dataset is observational, and the reported relationships cannot establish causal effects. Disease outbreaks or increased treatment demand may themselves prompt changes in hygiene or separation practices, creating potential reverse causation. Unmeasured disease severity, veterinary prescribing decisions and other farm characteristics may also influence both recorded biosecurity and AMU. Farm-specific intercepts and grouped predictive validation do not resolve these sources of confounding or establish temporal precedence.

Second, the biosecurity instrument was developed for within-farm monitoring and decision support. The author-confirmed codebook documents its parameter-specific definitions and intended coding direction, but the instrument has not been formally validated against an external reference standard. Inter-observer reliability and measurement equivalence between farms remain unestablished. Numeric slopes and composite scores also introduce assumptions about code-step linearity and comparability across items; the categorical sensitivity analyses address some of these modelling assumptions without validating the underlying measurements.

Third, monthly assessment and aggregation may obscure changes in disease events, management actions and antimicrobial decision-making within each month. One biosecurity assessment was linked to each analytical unit, and repeated substance-level scores did not require modal aggregation or tie-breaking. Nevertheless, the month-level export did not establish the precise ordering of assessment, illness and treatment. Its observed coverage also did not establish whether absent farm months represented zero use, non-reporting or other gaps. All available AMU values were positive, so the study could not characterise the distinction between any antimicrobial use and no use.

Fourth, the retained outcome was a mass-based sum-of-ratios AMU index. The recorded animal count denominator was identical across substances within an analytical unit, but the same animals could contribute repeatedly to recorded counts. It was therefore not a verified unique animal population denominator. Differences in dosage, potency, route and duration also mean that equal masses across substances need not represent equivalent treatment intensity. The available export did not support reconstruction of a treatment frequency or DDDvet-based endpoint.

Fifth, the small and unevenly contributing farm sample constrained cluster-robust inference, influence assessment and predictive validation. Sparse code 3 support and the post hoc restrictions further limited interpretation of some contrasts. Agreement between overlapping unfiltered and filtered cohorts did not constitute independent replication. Finally, explainability methods improved transparency about fitted models but did not remove confounding, measurement error or limitations in exposure support.

Despite these limitations, the study provides a transparent framework for examining recorded biosecurity–AMU associations in livestock systems. The combined workflow distinguished adjusted associations, sensitivity to analytical choices, prediction in held-out farms and explanation of fitted models. Recorded separation contrasts showed model-dependent associations with the AMU index, while hygiene indicators described overlapping aspects of the management environment. The practical contribution is to identify patterns and measurement needs for further investigation while showing where the available data do not support stronger conclusions. Consideration of production stage, animal separation, hygiene and farm context together remains consistent with the broader antimicrobial stewardship literature, but this study does not establish the AMU reduction achievable through a particular intervention.

## 5. Conclusions

This study described substantial variation in the AMU index across farms and production-stage units and identified model-dependent associations with recorded animal separation conditions. Code 2 and code 3 contrasts were positive in the main continuous outcome models, but their statistical support depended on hygiene representation, farm composition and uncertainty estimation. The code 2 association in the primary unfiltered separate-variable model was sensitive to the uncertainty procedure, while sparse support limited interpretation of the highest separation category. These findings did not establish a monotonic dose–response, a biological threshold or a causal management effect.

Continuous outcome models with farm-clustered uncertainty provided the primary inferential basis. Cohort restrictions, filter decomposition, farm-influence diagnostics and wild-cluster tests assessed sensitivity rather than replacing the unfiltered analysis. Secondary Firth models described elevated binary point estimates for separation codes 2 and 3, but bootstrap instability and wide intervals prevented precise effect quantification.

Farm-held-out validation did not establish consistent predictive improvement from the recorded biosecurity variables beyond production-stage information and simple baselines. SHAP provided exploratory explanations of the specified XGBoost models and did not independently demonstrate causal importance or operational predictive utility.

The findings identify patterns worth investigating within an explicitly documented analytical framework. Further work evaluating the reliability and external validity of the scoring instrument, improving temporal and treatment denominator information, and incorporating broader independent farm coverage is needed before causal or operational prediction claims can be made. Production stage, separation practices, hygiene and farm context remain relevant dimensions for future investigation, while the present data do not quantify the effects of changing those practices.

## Figures and Tables

**Table 1:** Analytical populations used in the main analyses. Batches denote Farm × Year × Month × Age Group units. Exclusions are expressed relative to the unfiltered cohort.

Cohort	Batches	Farms	Excluded Batches	Excluded Batches (%)	Main Exclusions	Analytical Role
Unfiltered cohort	1110	18	0	0	None	Primary analytical population
Filtered cohort	939	13	171	15.4	Selected production-stage categories and five anonymized farms	Restricted-population sensitivity cohort

**Table 2:** Primary continuous outcome estimates for Animal Population Separation across cohort and biosecurity specifications. Confidence intervals and conventional *p*-values use farm-clustered CR1 covariance with a t(G − 1) reference distribution. *p* < 0.05 was considered significant.

Cohort	Biosecurity Specification	Separation Contrast	β	95% CI	*p*-Value	Batches	Farms
Unfiltered	Composite	Code 1 vs. 0	−0.187	−0.510 to 0.135	0.237	1110	18
Unfiltered	Composite	Code 2 vs. 0	0.045	−0.149 to 0.239	0.629	1110	18
Unfiltered	Composite	Code 3 vs. 0	0.343	−0.083 to 0.768	0.108	1110	18
Unfiltered	Separate-variable	Code 1 vs. 0	−0.048	−0.332 to 0.236	0.725	1110	18
Unfiltered	Separate-variable	Code 2 vs. 0	0.205	0.013 to 0.397	0.037	1110	18
Unfiltered	Separate-variable	Code 3 vs. 0	0.337	−0.157 to 0.830	0.168	1110	18
Filtered	Composite	Code 1 vs. 0	−0.202	−0.542 to 0.138	0.221	939	13
Filtered	Composite	Code 2 vs. 0	0.112	−0.073 to 0.298	0.211	939	13
Filtered	Composite	Code 3 vs. 0	0.374	−0.096 to 0.845	0.109	939	13
Filtered	Separate-variable	Code 1 vs. 0	−0.073	−0.350 to 0.203	0.574	939	13
Filtered	Separate-variable	Code 2 vs. 0	0.272	0.125 to 0.419	0.002	939	13
Filtered	Separate-variable	Code 3 vs. 0	0.264	−0.270 to 0.799	0.303	939	13

**Table 3:** (**A**) Farm-held-out predictive performance for procedures that selected model family and hyperparameters within training folds. All metrics are pooled across held-out predictions. (**B**) Farm-held-out predictive performance for procedures that selected model family and hyperparameters within training folds. All metrics are pooled across held-out predictions, corresponding benchmarks.

(**A**)							
**Cohort**	**Validation**	**Biosecurity + Age RMSE**	**Biosecurity + Age MAE**	**Biosecurity + Age R^2^**	**Biosecurity + Age + Calendar RMSE**	**Cohort**	**Validation**
Unfiltered	Nested grouped CV	0.780	0.635	−0.101	0.793	Unfiltered	Nested grouped CV
Unfiltered	Leave-one-farm-out	0.810	0.647	−0.187	0.807	Unfiltered	Leave-one-farm-out
Filtered	Nested grouped CV	0.803	0.654	−0.151	0.804	Filtered	Nested grouped CV
Filtered	Leave-one-farm-out	0.782	0.644	−0.094	0.782	Filtered	Leave-one-farm-out
(**B**)							
**Cohort**	**Validation**	**Inner-Selected Age-Only RMSE**	**Training-Fold Mean RMSE**	**Training-Fold Stage-Mean RMSE**	**Cohort**	**Validation**	**Inner-Selected Age-Only RMSE**
Unfiltered	Nested grouped CV	0.765	0.763	0.743	Unfiltered	Nested grouped CV	0.765
Unfiltered	Leave-one-farm-out	0.770	0.759	0.742	Unfiltered	Leave-one-farm-out	0.770
Filtered	Nested grouped CV	0.786	0.771	0.766	Filtered	Nested grouped CV	0.786
Filtered	Leave-one-farm-out	0.789	0.766	0.764	Filtered	Leave-one-farm-out	0.789

## Data Availability

The datasets generated and analysed during the current study are not publicly available due to the inclusion of sensitive commercial information from producers but are available from the corresponding author on reasonable request.

## Supplementary Materials

The following supporting information can be downloaded at: https://www.mdpi.com/article/10.3390/vetsci13090971/s1, Figure S1: Exploratory SHAP summary from compatible tree-based model.; Figure S2: Within-farm temporal AMU variation using anonymised farm IDs.; Figure S3: Hygiene PCA score structure colored by AMU.; Figure S4: The leave-one-farm-out inferential influence diagnostics.; Figure S5: Hygiene PCA loading-vector map.; Figure S6: Leave-one-farm-out ML transportability sensitivity-filtered cohort. Table S1: Internal biosecurity codebook: parameter-specific category definitions and analytical codes; Table S2: Cohort-restriction sensitivity analysis of associations between Animal Population Separation and the log-transformed AMU index; Table S3: Outcome support and farm representation across Animal Population Separation categories in the unfiltered and filtered cohorts; Table S4: Production stage-specific support for Animal Population Separation categories: analytical unit counts and farm representation by cohort; Table S5: Exploratory calendar-adjusted associations between Animal Population Separation and the log-transformed AMU index in suckling piglets; Table S6: Hyperparameter search grids and initial estimator configurations for the machine learning regression models; Table S7: Pooled and mean farm-level predictive performance of regression models and baselines across feature sets and farm-held-out validation schemes; Table S8: Sensitivity of Animal Population Separation estimates to alternative hygiene composites derived using principal component analysis, factor analysis and weighted averaging; Table S9: Wild-cluster bootstrap tests of individual and joint Animal Population Separation contrasts across cohorts and biosecurity specifications; Table S10: Sensitivity of Animal Population Separation estimates to categorical hygiene coding, calendar adjustment and farm-specific intercepts; Table S11: Observation-level TreeSHAP contributions, expected outputs and predictions from XGBoost models evaluated on held-out farms in the unfiltered cohort.

## Abbreviations

The following abbreviations are used in this manuscript:

AI	Artificial intelligence
AMR	Antimicrobial resistance
AMU	Antimicrobial use
AUC	Area under the curve
CV	Cross-validation
FAO	Food and Agriculture Organization of the United Nations
MAE	Mean absolute error
ML	Machine learning
PCA	Principal component analysis
ROC	Receiver operating characteristic
SHAP	SHapley Additive exPlanations
t-SNE	t-distributed stochastic neighbor embedding
XGBoost	Extreme Gradient Boosting
